# SOX17 Is a Critical Specifier of Human Primordial Germ Cell Fate

**DOI:** 10.1016/j.cell.2014.12.013

**Published:** 2015-01-15

**Authors:** Naoko Irie, Leehee Weinberger, Walfred W.C. Tang, Toshihiro Kobayashi, Sergey Viukov, Yair S. Manor, Sabine Dietmann, Jacob H. Hanna, M. Azim Surani

**Affiliations:** 1 Wellcome Trust Cancer Research UK Gurdon Institute, Tennis Court Road, University of Cambridge, Cambridge CB2 1QN, UK; 2 Department of Physiology, Development and Neuroscience, Downing Street, University of Cambridge, Cambridge CB2 3EG, UK; 3 Wellcome Trust-Medical Research Council Stem Cell Institute, Tennis Court Road, University of Cambridge, Cambridge CB2 3EG, UK; 4 The Department of Molecular Genetics, Weizmann Institute of Science, Rehovot 76100, Israel

## Abstract

Specification of primordial germ cells (PGCs) marks the beginning of the totipotent state. However, without a tractable experimental model, the mechanism of human PGC (hPGC) specification remains unclear. Here, we demonstrate specification of hPGC-like cells (hPGCLCs) from germline competent pluripotent stem cells. The characteristics of hPGCLCs are consistent with the embryonic hPGCs and a germline seminoma that share a CD38 cell-surface marker, which collectively defines likely progression of the early human germline. Remarkably, SOX17 is the key regulator of hPGC-like fate, whereas BLIMP1 represses endodermal and other somatic genes during specification of hPGCLCs. Notable mechanistic differences between mouse and human PGC specification could be attributed to their divergent embryonic development and pluripotent states, which might affect other early cell-fate decisions. We have established a foundation for future studies on resetting of the epigenome in hPGCLCs and hPGCs for totipotency and the transmission of genetic and epigenetic information.

## Highlights

• A defined model for hPGCLC specification from germline-competent hESCs
• Expression profiles of hPGCLCs match with authentic hPGCs
• SOX17 is the key regulator of hPGCLC
• CD38 glycoprotein is a cell-surface marker of the human germline

## Graphical Abstract

**Figure:**
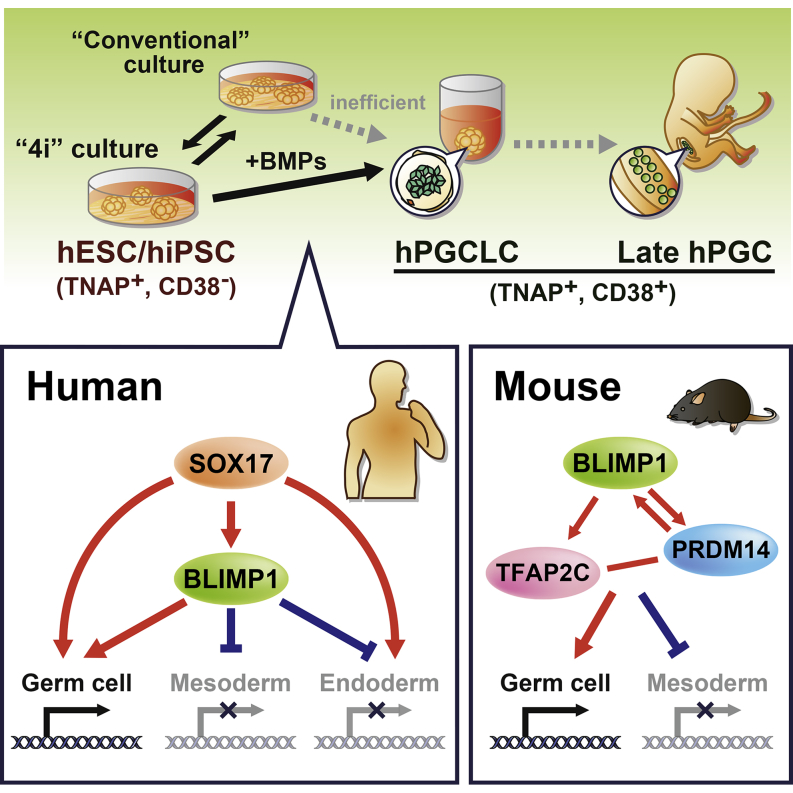

## Teaser

Different from that in mice, SOX17 is the key regulator for human primordial germ cell specification, suggesting fundamental differences between early mouse and human development.

## Introduction

Primordial germ cells (PGCs) are the precursors of sperm and eggs, which generate the totipotent state. The genetic basis of mammalian PGC specification was first established in mice (Saitou et al., 2002; Ohinata et al., 2005; Hayashi et al., 2007), which are specified from postimplantation epiblast cells on embryonic day (E)6.25 in response to bone morphogenetic protein 4 (BMP4) (Lawson et al., 1999). Subsequently, ∼35 founder PGCs are detected at E7.25. Similar studies on human PGCs (hPGCs) would require E9–E16 embryos, which is not practicable. However, embryonic hPGCs at approximately week 5 to 10 of development, which correspond to mouse PGCs at E10.5–E13.5, can in principle be examined (Leitch et al., 2013). These cells retain characteristic of PGCs while they undergo resetting of the epigenome and global DNA demethylation (Hackett et al., 2012).

In mice, BMP4 induces expression of BLIMP1 (encoded by *Prdm1*) and PRDM14 in the postimplantation epiblast at E6.25; together with AP2γ (encoded by *Tfap2c*), a direct target of BLIMP1, they induce PGC fate (Magnúsdóttir et al., 2013; Nakaki et al., 2013). The tripartite genetic network acts combinatorially to repress somatic genes, induce expression of PGC genes, such as *Nanos3*, reinduce pluripotency genes, and initiate the epigenetic program (Hackett et al., 2013; Magnúsdóttir and Surani, 2014). PGC-like cells (PGCLCs) can also be induced in vitro from naive pluripotent mouse embryonic stem cells (mESCs) after they acquire competence for germ cell fate after ∼48 hr culture in basic fibroblast growth factor (bFGF) and Activin A (Hayashi et al., 2011). These competent cells acquire PGC-like fate in response to either BMP4 signal or directly to *Blimp1*, *Prdm14*, and *Tfap2c*, which is similar to PGCs in vivo (Magnúsdóttir et al., 2013; Nakaki et al., 2013).

Human PGCLCs (hPGCLCs) have been generated at a low frequency by spontaneous differentiation of human ESCs (hESC) in vitro (Gkountela et al., 2013; Kee et al., 2009), but systematic studies to characterize and identify the key regulators of hPGCs remain to be elucidated. Because there are evident differences between the regulation of mouse and human pluripotent ESCs (Hackett and Surani, 2014; Nichols and Smith, 2009) and during their early postimplantation development (de Fellici, 2013; De Miguel et al., 2010; Irie et al., 2014), this might affect the mechanism and the role of the key regulators of hPGCLC specification (Imamura et al., 2014; Pera, 2013). Once the mechanism of hPGCLC specification is established, it could provide insights on the progression of the early human germline with reference to embryonic hPGCs and seminomas that originate from human germ cells in vivo and retain key characteristics of the lineage (Looijenga et al., 2014).

We have developed a robust approach for hPGCLC specification from germ cell competent hESCs/hiPSCs (Gafni et al., 2013). We show that SOX17, a critical transcription factor for endoderm lineages, is the earliest marker of hPGCLCs and is in fact the key regulator of hPGCLC fate, which is not the case in mice (Hara et al., 2009; Kanai-Azuma et al., 2002). BLIMP1 is downstream of SOX17, and it represses endodermal and other somatic genes during hPGCLC specification. Comparisons among hPGCLCs, embryonic hPGCs, and a seminoma indicate likely progression of the early human germline. These cells also exhibit CD38 cell surface marker, which is shared by cells with germ cell characteristics. We anticipate that genome editing approaches with our robust in vitro model for hPGCLC specification, combined with patient-specific human-induced pluripotent stem cells (hiPSCs), will lead to major advances in human germ cell biology, including on the unique germline-specific epigenetic program with potential consequences for subsequent generations.

## Results

### Generation of hPGCLCs from Embryonic Stem Cells

**Figure S1:**
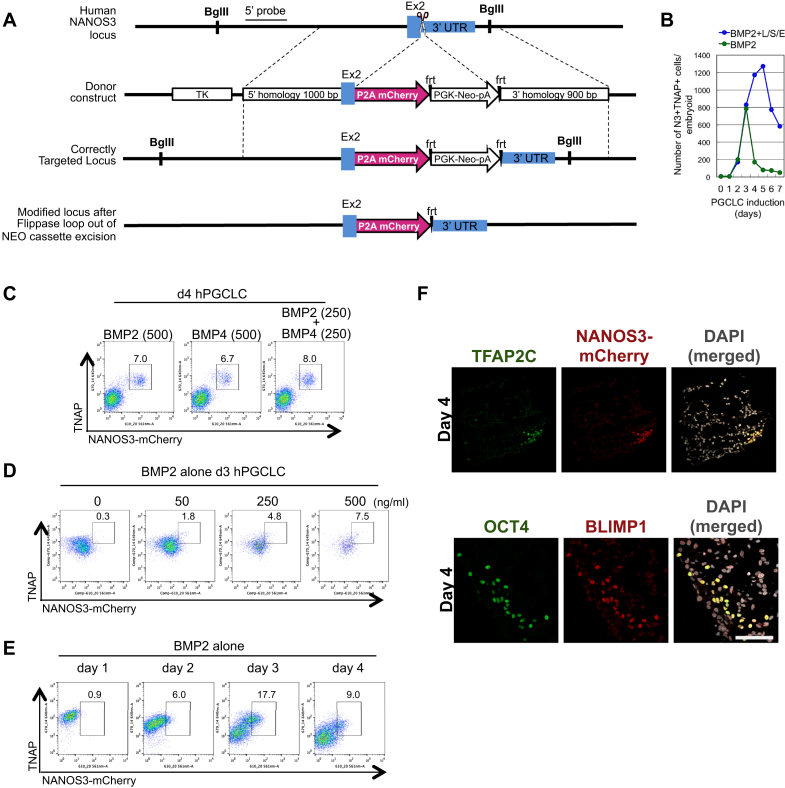
Generation of NANOS3-mCherry Reporter Knockin hESC Lines and hPGCLC Differentiation, Related to Figure 1 (A) Targeting strategy of generation of NANOS3-mCherry knock-in reporter hESC lines. P2A-mCherry sequence in frame with the last exon of the human NANOS3 gene was inserted. We have generated plasmids encoding TALEN molecules specific to the region covering NANOS3 stop codon. Scissors indicate TALEN cutting site. Southern blot was performed with 5′ external probe (5′ probe) and the BglII restriction enzyme sites. Correct targeting and loop-out of resistance cassette was conducted in three independent human ESC lines (WIS2, LIS1 and WIBR3). (B) Number of the NANOS3-mCherry/TNAP positive cells per embryoid during PGCLC induction with BMP2, human LIF, SCF and EGF (BMP2+L/S/E) or BMP2 alone. (C) FACS analysis for NANOS3-mCherry and TNAP positive population using WIS2-NANOS3-mCherry cell line after 4 days of hPGCLC induction by BMP2 (500 ng/ml), BMP4 (500 ng/ml) or BMP2 (250 ng/ml) and BMP4 (250 ng/ml) together with human LIF, SCF, EGF and ROCK inhibitor. Numbers show the percentage of the TNAP/NANOS3 double positive population in the boxes. (D) FACS analysis for NANOS3-mCherry and TNAP positive population using WIS2-NANOS3-mCherry cell line after 3 days of hPGCLC induction with BMP2 (50, 250 and 500 ng/ml) or without BMP2 (0) in the presence of ROCK inhibitor. (E) FACS analysis of NANOS3-mCherry and TNAP positive population from WIS2-NANOS3-mCherry cell line with PGCLC induction with BMP2 alone from day 1 to day 4. (F) Immunofluorescence of TFAP2C and NANOS3-mCherry, and OCT4 and BLIMP1 on day 4 embryoids. Scale bar = 70 μm.

First, we generated three independent hESC lines (WIS2 and LIS1 male hESC and WIBR3 female hESC line) (Gafni et al., 2013) with a NANOS3-mCherry knockin reporter (Figure S1A available online), a highly conserved PGC-specific gene (Gkountela et al., 2013; Julaton and Reijo Pera, 2011). These hESCs maintained in bFGF and responded to BMP2/BMP4 with ∼0%–5% NANOS3-mCherry positive putative hPGCLCs at day 4 (see Figure 7A). Like hESC, mouse epiblast stem cells (mEpiSC) also respond poorly to specification of PGCLCs (Hayashi and Surani, 2009). In contrast, epiblast-like cells (EpiLCs) derived from naive mESCs have a significant potential for germ cell fate (Hayashi et al., 2011). However, the approach used for mouse ESCs did not confer competence for germline fate on hESCs.

**Figure 1:**
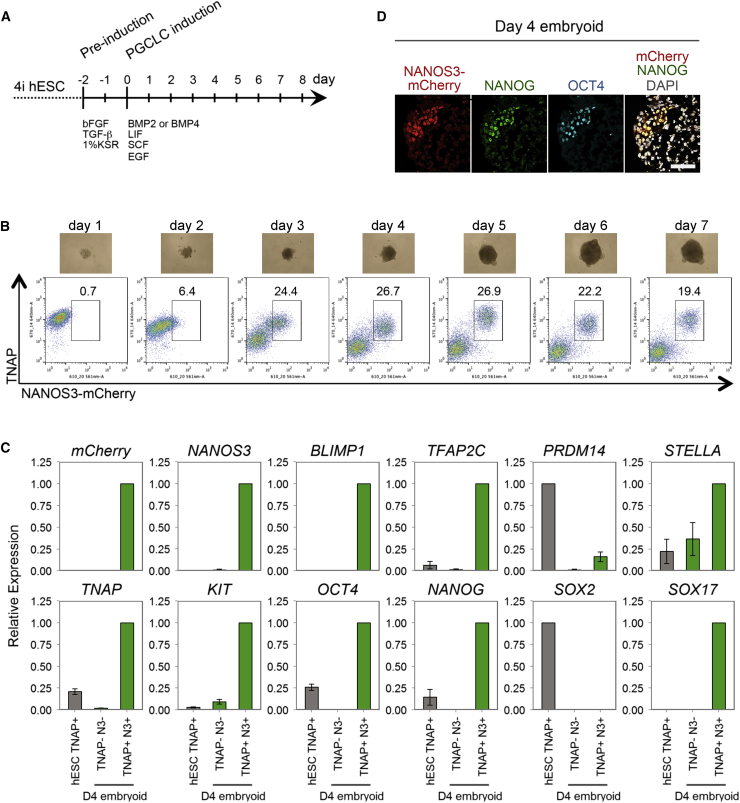
Specification of hPGCLCs from Human Embryonic Stem Cells (A) Schematic protocol for hPGCLCs specification from hESCs. (B) Development of day 1–7 embryoids derived from WIS2-NANOS3-mCherry hESCs. Top row: images of embryoids. Bottom row: FACS analysis of the dissociated embryoids with anti-TNAP-Alexa Fluor 647 and NANOS3-mCherry to detect hPGCLCs. (C) Expression analysis by RT-qPCR of TNAP-positive 4i hESCs (hESC TNAP+), TNAP/NANOS3-mCherry-positive hPGCLCs (TNAP+N3+), and the remaining cells (TNAP-N3-) of day 4 embryoids (D4 embryoid). Relative expression levels are shown with normalization to *β−ACTIN*. Error bars indicate mean ± SD from three independent biological replicates. (D) Immunofluorescence of a day 4 embryoid showing coexpression of NANOS3-mCherry, NANOG, and OCT4 in hPGCLCs. Scale bar, 66 μm.

Next, we tested hESC-NANOS3-mCherry cells that were maintained in four-inhibitor-containing medium with LIF, bFGF, and TGFβ (adopted and modified from NHSM conditions; see Experimental Procedures), henceforth called “4i” medium, which endows the cells with a distinct pluripotent state (Gafni et al., 2013). These hESCs were then cultured for 2 days in bFGF, TGFβ, and 1% KSR medium, and thereafter, 2,000–4,000 cells were cultured in low-attachment well in the presence of BMP2 or BMP4, LIF, stem cell factor (SCF), epidermal growth factor (EGF), and Rho-kinase (ROCK) inhibitor to induce hPGCLCs (Hayashi et al., 2011; Watanabe et al., 2007) (Figure 1A). These cells aggregated to form embryoid bodies (henceforth called embyoids) and responded within 3 days with significant expression of NANOS3-mCherry and tissue-nonspecific alkaline phosphatase (TNAP), a PGC and pluripotency marker in humans and mice (Figure 1B). The intensity of the NANOS3-mCherry reporter increased progressively until day 4–5, resulting in ∼27% of NANOS3/TNAP double-positive putative hPGCLCs (Figures 1B and S1B). Similar to mice, hPGCLCs do not proliferate significantly after 5 days under these conditions (Hayashi et al., 2011). The response was highly reproducible in three independent male and female NANOS3-mCherry hESC lines. Both BMP2 and/or BMP4 (with LIF, SCF, and EGF) were effective in inducing hPGCLC (Figure S1C) in a dose-dependent manner in the range of 50–500 ng/ml (Figures S1D and S1E).

**Figure 2:**
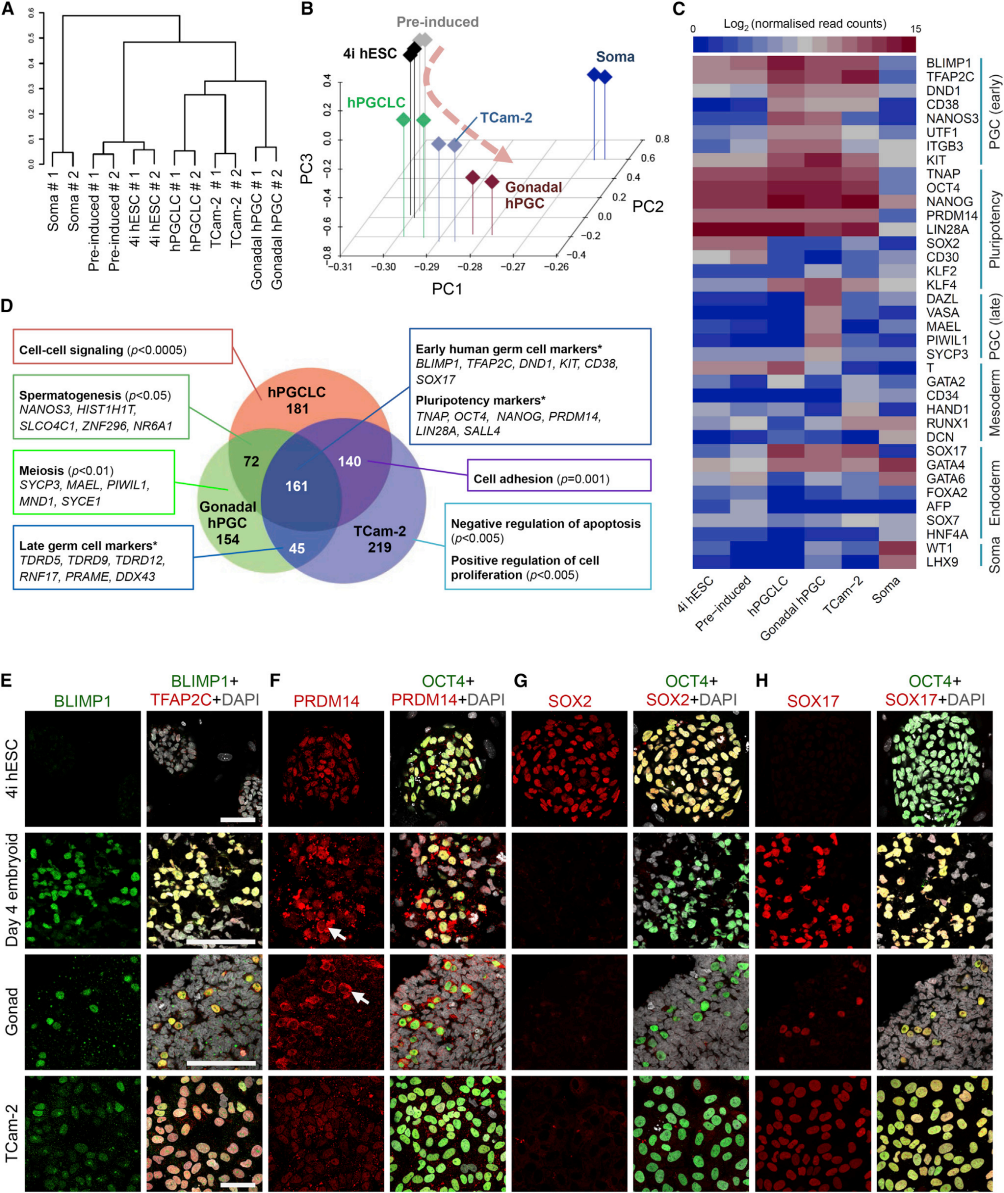
hPGCLC Shares Transcriptional Profile with Human Embryonic PGCs and TCam-2 Seminoma (A) Unsupervised hierarchical clustering (UHC) of gene expression in 4i hESC, preinduced cells (Pre-induced), day 4 hPGCLCs (hPGCLC), gonadal hPGC, TCam-2, and gonadal somatic cell (Soma). RNA-seq was performed on two biological replicates (#1 and #2) for each cell type. (B) PCA of RNA-seq data. Arrowline indicates potential germline progression from 4i hESC to hPGCLC and gonadal hPGC. (C) Heat map of gene expression of key PGC-associated genes (early and late) and of pluripotency, mesoderm, endoderm, and gonadal somatic (Soma) markers. (D) Venn diagram illustrates common and differentially expressed genes. Significantly upregulated genes in hPGCLC, gonadal hPGC, and TCam-2 (with log2 (fold change) > 3 and adjusted p value < 0.05 versus gonadal Soma, respectively) were compared. Representative genes that were exclusive to each category are indicated. Text boxes indicate gene ontology biological processes (BP) terms that were significantly enriched as indicated by p values. Asterisk denotes custom categories absent from BP annotation. (E–H) Immunofluorescence analysis for (E) BLIMP1, (F) PRDM14, (G) SOX2, and (H) SOX17 on 4i hESCs (top row), day 4 hPGCLC embryoids (second row), human week 7 male gonad (third row), and TCam-2 (bottom row). Samples were counterstained with TFAP2C or OCT4 to identify hPGCLCs in embryoids and hPGCs in embryonic gonad. Arrows indicate cytoplasmic enrichment of PRDM14 (F). Scale bars, 70 μm.

The NANOS3/TNAP double-positive putative hPGCLCs also expressed key PGC genes, including *NANOS3*, *BLIMP1*, *TFAP2C*, *STELLA*, *TNAP*, *KIT*, *OCT4*, and *NANOG*, as well as *PRDM14*, albeit with reduced levels compared to hESC (Figure 1C). Remarkably, *SOX17* was significantly upregulated, whereas *SOX2* was downregulated in the putative hPGCLCs that reflects their expression in embryonic hPGCs and seminomas (de Jong et al., 2008; see Figure 2), which is not the case in mouse PGCs. Immunofluorescence confirmed that NANOS3-mCherry expression coincided with OCT4, NANOG, and TFAP2C in day 4 embryoids (Figures 1D and S1F), as did OCT4 with BLIMP1 (Figure S1F). This suggests that the NANOS3-mCherry-positive cells are very likely nascent germ cells.

### RNA-Seq Analysis of hPGCLCs: Comparison with hPGCs and Seminoma

We carried out RNA sequencing (RNA-seq) on NANOS3/TNAP double-positive cells from day 4 embryoids and compared them with the gonadal hPGCs from week 7 male human embryos (Carnegie stage 18/19), which are equivalent to mouse ∼E12.5–E13.5 PGCs (Leitch et al., 2013). These hPGCs retain key characteristics of earlier hPGCs but, consistent with their more advanced state, expresses later germ cell markers such as VASA and DAZL. We also included TCam-2, a human seminoma that originates from the germline in vivo (Looijenga et al., 2014).

**Figure S2:**
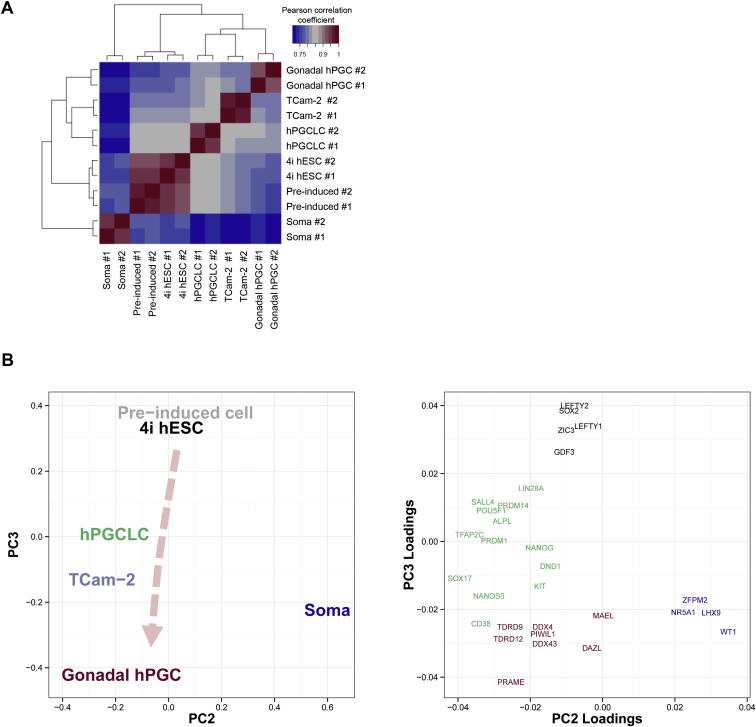
Global Transcriptome Analysis of hPGCLC, Related to Figure 2 (A) Pearson correlation heat map of gene expression (log_2_(normalized read counts)) in various samples with unsupervised hierarchical clustering. The color key indicates the correlation coefficient. (B) Two-dimensional principal components analysis of gene expression (PC3 against PC2) of the indicated samples (left panel). A corresponding loadings plot indicates the weight of various genes on PC2 and PC3 (right panel). Gene names are color-coded to illustrate association with pluripotency (black), early germ cell development (green), late germ cell development (red) and gonadal somatic cell development (blue). Arrowline indicates potential germline progression from 4i hESC via hPGCLC to gonadal hPGC.

Unsupervised hierarchical clustering of global gene expression showed that the hPGCLCs clustered with hPGCs and TCam-2, whereas 4i hESCs and preinduced cells (4i hESCs treated with bFGF and TGFβ for 2 days) clustered together in another branch away from gonadal somatic cells (soma) (Figure 2A). Consistently, hPGCs were globally more related to hPGCLCs (Pearson correlation coefficient [*r*] = 0.85) and TCam-2 (*r* = 0.818) than to 4i hESCs (*r* = 0.799) and preinduced cells (*r* = 0.773) (Figure S2A).

**Figure 3:**
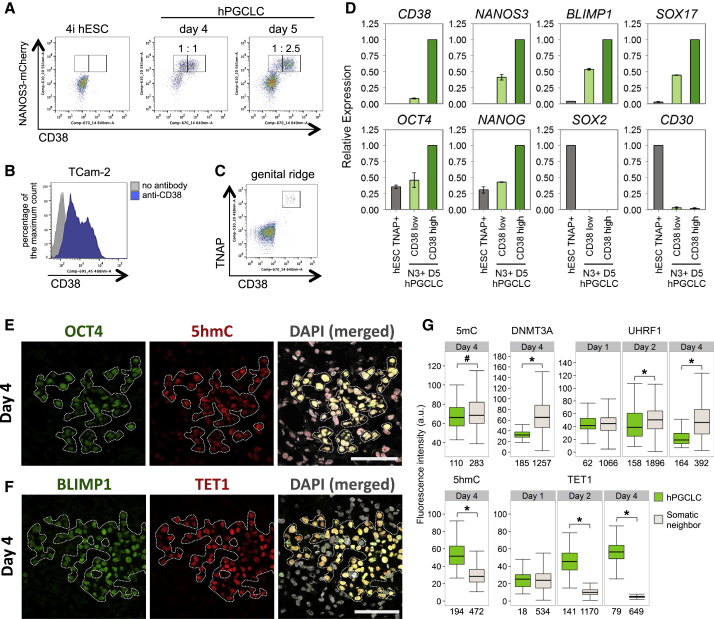
CD38 Expression in Human Germ-Cell-Related Cells and Epigenetic Changes in hPGCLCs (A) FACS analysis of NANOS3-mCherry and CD38 on WIS2-NANOS3-mCherry cell line cultured in 4i medium and on day 4 and 5 embryoids following hPGCLC induction. Ratios of CD38 low and high expression in the NANOS3-mCherry-positive cells are indicated. (B) FACS histogram of CD38 low and high populations in TCam-2. (C) FACS analysis of CD38 and TNAP on genital ridges isolated from a week 6 human embryo. (D) Expression analysis by RT-qPCR for FACS-sorted TNAP-positive 4i hESCs (TNAP+ hESC) and CD38 low or high/NANOS3-mCherry day 5 hPGCLCs. Relative expression levels are shown with normalization to *β−ACTIN*. Error bars indicate mean ± SD from two independent biological replicates. (E and F) Immunofluorescence analysis for 5hmC (E) and TET1 (F) on day 4 embryoids cryosection. OCT4 or BLIMP1 were used to identify hPGCLCs (highlighted). Scale bars, 50 μm. (G) Quantification of immunofluorescence intensity of various epigenetic marks/modifiers in hPGCLCs and somatic neighbors in day 1–4 embryoids (see also Figures S3A–S3C). For UHRF1, only KI-67-positive (proliferating) cells were used for quantification. Numbers below each box denotes number of cells analyzed. Black central line represents the median, boxes and whiskers represent the 25^th^ and 75^th^, and 2.5^th^ and 97.5^th^ percentiles, respectively. Wilcoxon signed-rank test was used to test for statistical significance. #p < 0.05; ^∗^p < 0.0001.

A heat map of mRNA expression revealed that hPGCLCs and gonadal hPGCs shared expression of early PGCs (*BLIMP1*, *TFAP2C*, *DND1*, *NANOS3*, *UTF1*, *ITGB3*, and *KIT*) and pluripotency genes (*TNAP*, *OCT4*, *NANOG*, *PRDM14*, and *LIN28A*) but with a notable lack of *SOX2* expression (Figure 2C). Early mesoderm marker *T* was detected in hPGCLCs (Figure 2C), as in mouse early PGCs (Aramaki et al., 2013). Interestingly, expression of two endodermal genes, *SOX17* and *GATA4*, was detected in hPGCLCs, embryonic hPGCs, and TCam-2, which are absent in the mouse germline. Notably, we identified *CD38* expression in hPGCLCs/hPGCs and TCam-2, but not in hESCs or soma (Figures 2C and see also Figures 3A–3C). Overall, hPGCLCs indeed have germ cell characteristics consistent with hPGCs. Late germ cell markers, however, including *DAZL*, *VASA*, and *MAEL*, were only detected in hPGCs (Figure 2C). TCam-2 gene expression was similar to hPGCLCs, albeit with lower expression levels of *NANOS3*, *ITGB3*, and *T* and upregulation of a few somatic genes, e.g., *HAND1* and *RUNX1*. Immunofluorescence analysis validated the expression of BLIMP1, TFAP2C, and OCT4 in hPGCLCs/hPGCs and TCam-2 (Figures 2E–2H). Interestingly, PRDM14 showed nuclear localization in the majority of hPGCLCs but was predominantly enriched in the cytoplasm of hPGCs (Figure 2F). Importantly, although SOX2 was undetectable, there was significant expression of SOX17 in hPGCLCs, hPGCs, and TCam-2 (Figures 2G and 2H).

Given the similarities of hPGCLCs, hPGCs, and TCam-2, a three-way Venn diagram was plotted to investigate their relationships (Figure 2D). Out of 972 highly upregulated genes compared to soma (Table S1), the three germline-related cell types shared expression of 161 genes, including pluripotency and germline-specific genes: *BLIMP1*, *TFAP2C*, *CD38*, *SOX17*, *OCT4*, and *NANOG* (Figure 2D). Gene ontology (GO biological process) analysis revealed (Table S1) that hPGCLCs from male cell line and male gonadal hPGCs were commonly enriched in “spermatogenesis” genes—for example, *NANOS3* and *HIST1H1T*—whereas meiosis-related *SYCP3*, *MAEL*, and *PIWIL1* genes were upregulated only in embryonic hPGCs (Figures 2C and 2D). Interestingly, TCam-2 and hPGCs revealed expression of a number of late germ cell markers, including Tudor-domain-containing *TDRD5*, *TDRD9*, and *TDRD12* genes, which have been implicated in PIWI-interacting RNA biogenesis pathway (Shoji et al., 2009) (Figure 2D). As expected, TCam-2 showed characteristics associated with cancer cells, including genes that promote cell proliferation with suppression of apoptosis genes (Figure 2D). Altogether, hPGCLCs, TCam-2, and hPGCs share key germ cell characteristics and expressed the core germ cell genes, including *CD38*, whereas the differentially expressed genes reflected their corresponding stages of development and cell identity.

Principal component analysis (PCA) further illustrates the relationships between the different cell types. PCA reduces dimensionality of whole-genome expression data by transforming into principal components (PCs), in which the variance within the dataset is maximal. A three-dimensional (3D) PCA plot of the first three PCs showed that the 4i hESC, soma, and hPGC-related cells (hPGCLCs, gonadal hPGCs, and TCam-2) settled at three discrete positions (Figure 2B). In particular, hPGCLCs, TCam-2, and gonadal hPGCs aligned together at the lower extreme of PC2, whereas 4i hESCs and preinduced cells formed a distinct cluster with medium PC2 scores and soma at the upper extreme (Figures 2B and S2B). The relative contributions (weights) of key germ cell, pluripotency, and gonadal somatic genes to PC2 and PC3 were plotted as two-dimensional (2D) loading plot alongside a corresponding 2D PCA plot (Figure S2B). Indeed, the weights of germ cell, pluripotency, and somatic genes highly overlap with the position of germ-cell-related cell types, hESCs, and soma, respectively. Germ-cell-related genes, such as *SOX17*, *CD38*, and *NANOS3* loaded heavily for lower extreme of PC2, where hPGCLCs, TCam-2, and gonadal hPGCs were aligned. There was a clear difference in weights of early germ cell genes (commonly expressed in hPGCLCs, TCam-2, and gonadal hPGCs—for example, *BLIMP1* and *TFAP2C*) and late germ cell genes (expressed only in gonadal hPGCs or TCam-2—for example, *VASA* and *DAZL*) on PC3, with the latter weighing more heavily toward low PC3 scores (Figure S2B). Notably, decreasing scores of PC3 reflected potential progression of germ cell development from hPGCLCs toward gonadal hPGCs, whereas TCam-2 aligned between hPGCLCs and gonadal hPGCs (Figures 2B and S2B).

Taken together, hPGCLCs demonstrate germ cell characteristics that are apparently en route to hPGCs, whereas our objective analysis placed TCam-2 in an intermediate position, which reflects their origin from hPGCs in vivo. Notably, hPGCLCs evidently represent the earliest stages of the human germ cell lineage, indicating that our in vitro model provides an important opportunity to explore the mechanism of hPGC specification, which is otherwise not possible because E9–E14 postimplantation human embryos are excluded from investigations. TCam-2 and other seminomas might, however, also serve as important in vitro models of human germ cell biology (Looijenga et al., 2014; Schafer et al., 2011).

### CD38: A Core Marker of Human Germ-Cell-Related Cells and Initiation of the Epigenetic Program

CD38, an established cell-surface glycoprotein on leukocytes, is a prognostic marker of leukemia (Malavasi et al., 2008). Surprisingly, we detected *CD38* expression in hPGCLCs, gonadal hPGCs, and TCam-2, but not in hESCs or gonadal somatic cells (Figure 2C). Indeed, fluorescence-activated cell sorting (FACS) analysis showed that CD38 is present on all the TNAP-positive embryonic hPGCs and on TCam-2 with some heterogeneity (Figures 3B and 3C). Although CD38 is absent on hESCs, ∼50% of the NANOS3-mCherry-positive hPGCLCs were CD38 positive on day 4 (Figure 3A), which increased to ∼70% by day 5 (Figure 3A). Interestingly, the NANOS3-mCherry/CD38 cells had higher expression of *NANOS3*, *BLIMP1*, *SOX17*, *OCT4*, and *NANOG* (Figure 3D). By contrast, hESCs and embryonic carcinoma cells exhibit *CD30* (also known as *TNFRSF8*) and SOX2 (Figures 3D and 2G) (Pallesen and Hamilton-Dutoit, 1988). Thus, CD38 and CD30 could potentially be used as additional markers of germ cell tumors in vivo (Figure 7D).

**Figure S3:**
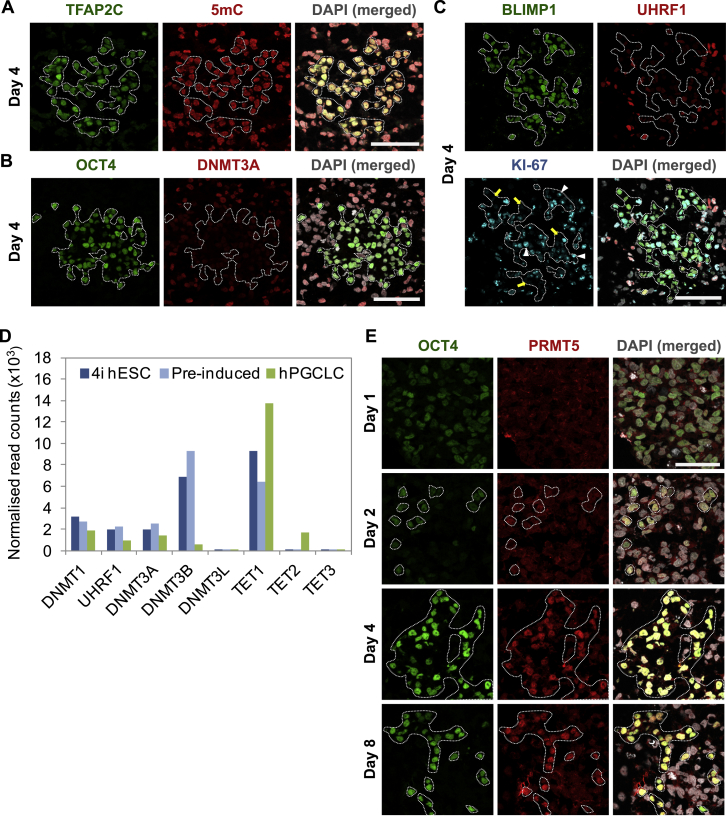
Immunofluorescence for Epigenetic Modifications and Modifiers in Embryoids, Related to Figure 3 (A–C) Immunofluorescence analysis was carried out for (A) 5-methylcytosine (5mC); (B) DNMT3A; (C) UHRF1 on day 4 embryoids. TFAP2C, OCT4 or BLIMP1 were used to counterstain for hPGCLCs. hPGCLCs were highlighted by white dashed lines. White arrowheads showed examples of KI-67-positive PGCLCs while yellow arrows showed the KI-67-negative PGCLCs (C). Scale bars = 50 μm. (D) Expression level of methylation related epigenetic modifiers from RNA-Seq dataset. Mean normalized read counts from two biological replicates were shown. (E) Immunofluoresence of PRMT5 on cryosections of embryoids collected at day 1, 2, 4 and 8 post-hPGCLC induction. hPGCLCs were counterstained with OCT4 as highlighted. Scale bar = 70 μm.

The RNA-seq of hPGCLC also revealed gene expression changes that indicate initiation of the epigenetic program with downregulation of *UHRF1*, *DNMT3A*, and *DNMT3B* and upregulation of *TET1* and *TET2* (Figure S3D). Notably, we found a significant increase in 5-hydroxymethylacytosine (5hmC) in hPGCLCs, which is consistent with an increase in the expression of TET1, an enzyme that converts 5-methylcytosine (5mC) to 5hmC (Figures 3E–3G), together with a small but significant decline in 5mC (Figures 3G and S3A). This indicates that, as in the mouse PGCs, loss of 5mC might be coupled with the conversion of 5mC to 5hmC (Hackett et al., 2013). At the same time, we detected a decline in the expression of de novo DNA methyltransferase 3A (DNMT3A) and UHRF1 in hPGCLCs compared to the neighboring somatic cells in the embryoids (Figures 3G, S3B, and S3C). UHRF1 targets DNMT1 to replication foci to confer maintenance of DNA methylation (Liu et al., 2013). The repression of UHRF1 in proliferating (KI-67-positive) hPGCLCs would allow DNA-replication-coupled loss of 5mC, which is analogous to the observations on the early mouse germline.

Taken together, day 4 hPGCLCs, which are the nascent human germ cells, already showed evidence for the initiation of epigenetic changes and DNA demethylation that are comparable to E8 mouse PGCs (Hackett et al., 2013). Notably, we also found that PRMT5, an arginine methylatransferase that was ubiquitously but weakly present in the cytoplasm of day 1 and 2 embryoids, showed enhanced expression in the nucleus of day 4–8 hPGCLCs (Figure S3E). This is a shared characteristic with ∼E8 mouse PGCs, hPGCs, and TCam-2 seminoma (Eckert et al., 2008). The translocation of PRMT5 to the nucleus is important for the suppression of transposable elements at the onset of DNA demethylation (Kim et al., 2014).

### Sequential Gene Expression during hPGCLC Specification in Embryoids

Having established similarities between hPGCLCs and the authentic hPGCs, we set out to investigate the mechanism of hPGCLC specification. First, for establishing the precise sequence of expression of the key hPGC-related genes at the resolution of single cells, we performed systematic time course analysis by immunofluorescence on day 1–8 embryoids after hPGCLC differentiation.

**Figure 4:**
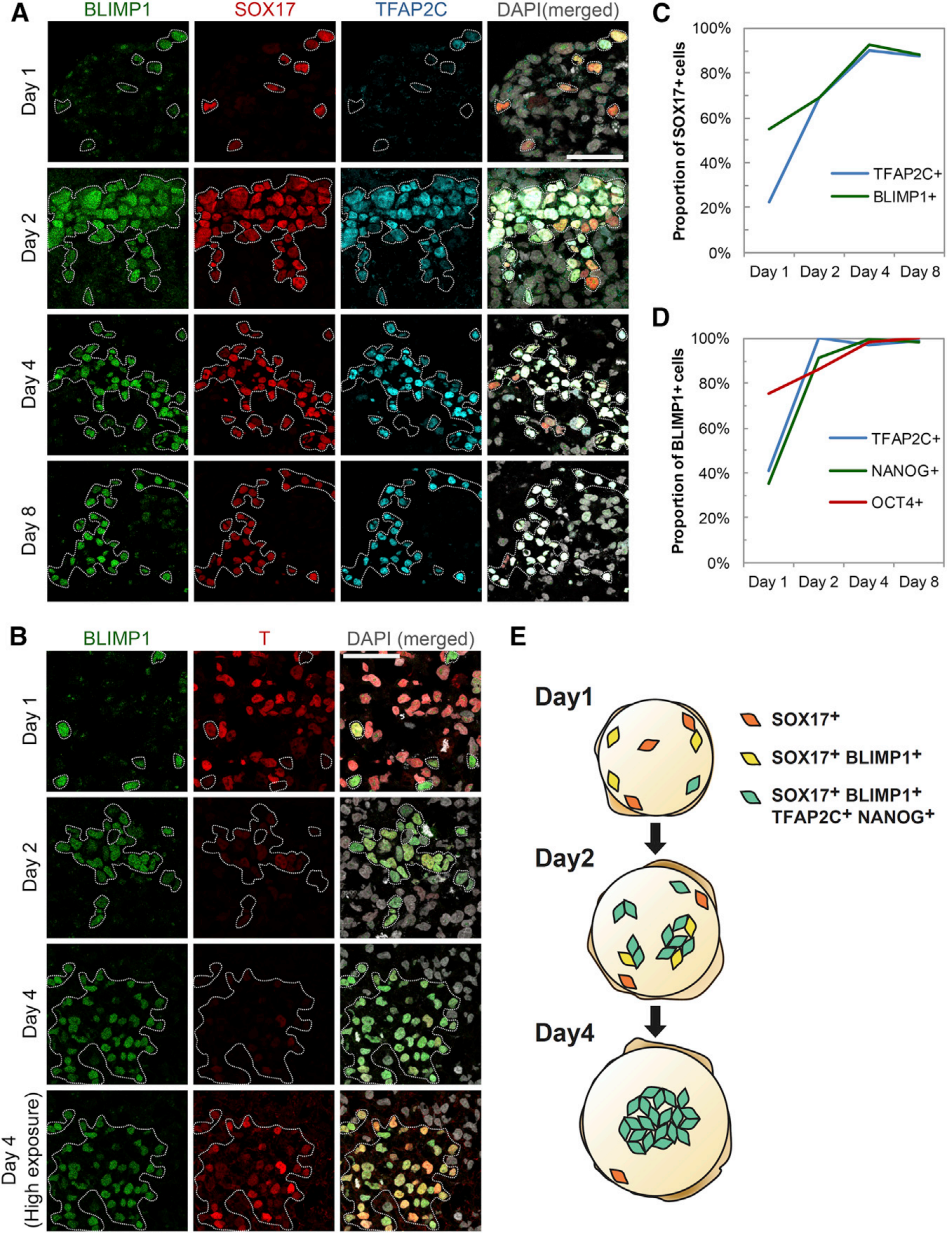
Sequential Expression of Germ-Cell-Related Transcription Factors in Single Cells during hPGCLC Specification (A and B) Immunofluorscence analysis for (A) BLIMP1, SOX17, and TFAP2C and (B) BLIMP1 and T in cryosections of day 1–8 embryoids after hPGCLC induction. Bottom row in (B) shows high exposure (digital) image of T, indicating low but specific expression in hPGCLC. SOX17-positive or BLIMP1-positive cells are highlighted. Scale bars, 50 μm. (C) Percentage of SOX17-positive (+) cells in day 1–8 embryoids that were also TFAP2C+ or BLIMP1+. Corresponds to data in Figure 4A. (D) Percentage of BLIMP1-positive (+) cells in day 1–8 embryoids that were TFAP2C+, NANOG+, or OCT4+. Corresponds to data in Figures 4A, S4A, and S4B. (E) Summary model for dynamics of hPGCLC specification in embryoids. SOX17-positive cells are first scattered in day 1 embryoids. They gain expression of BLIMP1, TFAP2C, and NANOG sequentially and form a cluster from day 2 onward until the formation of nascent hPGCLC.

On day 1, we first detected SOX17 in a few widely scattered cells throughout the embryoids (Figures 4A and 4E). Among the SOX17-positive (+) cells, 55% were also BLIMP1+, and 22% were TFAP2C+ (Figures 4A and 4C). However, all BLIMP1+ cells coexpressed SOX17, suggesting that SOX17 is upregulated before BLIMP1. The proportion of BLIMP1+ and TFAP2C+ cells increased to ∼70% on day 2 and to ∼90% on days 4–8 (Figures 4A and 4C). These triple-positive cells likely represent specified hPGCLCs, as they also coexpressed other key hPGC genes. However, ∼10% of single SOX17+ cells failed to undergo hPGCLC specification but persisted in day 4–8 embryoids. These may be aberrant cells or else may belong to other lineages.

Expression of T is of particular interest, as it signifies competence for germ cell fate in mice, and BMPs can induce it in hESCs (Bernardo et al., 2011; Yu et al., 2011). Notably, expression of T was high in the majority of cells on day 1, except for most of the BLIMP1+ cells (Figure 4B). By day 2, however, T was dramatically downregulated in most cells, although now the BLIMP1+ nascent hPGCLC retained low T expression, which persisted until at least day 4 (Figure 4B), consistent with the *T* transcripts detected by RNA-seq (Figure 2C). It is possible that BMP signaling may initially enhance expression of T in the embryoids (Bernardo et al., 2011), and it is from this population that hPGCLCs are specified, which reflects the events during mouse PGC induction (Aramaki et al., 2013).

**Figure S4:**
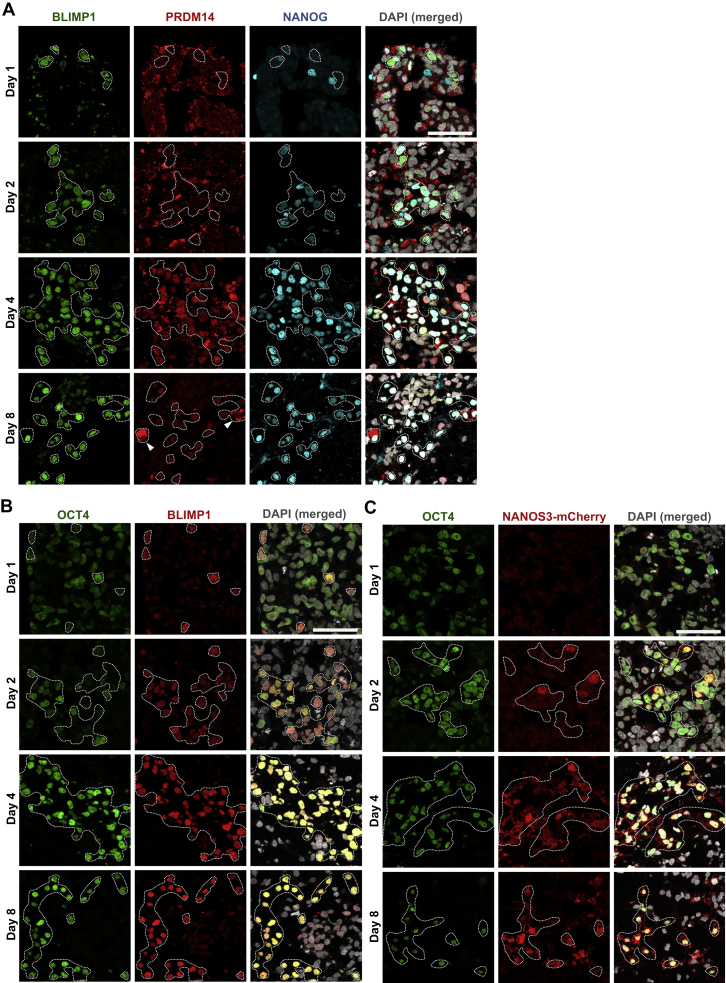
Sequential Expression of Germ-Cell-Related Genes during PGCLC Specification, Related to Figure 4 (A–C) Immunofluoresence of (A) PRDM14 and NANOG; (B) OCT4; and (C) NANOS3-mCherry on cryosections of day 1-8. hPGCLC were counterstained with BLIMP1 or OCT4 as highlighted. Arrowheads indicate enrichment of PRDM14 in cytoplasm. Scale bars = 70 μm.

Expression of OCT4 was low but widespread in the day 1 embryoids, including 75% of the BLIMP1+ cells (Figures S4B and 4D). Although the overall OCT4 expression declined dramatically in day 2 embryoids, it was strongly expressed in ∼86% of the BLIMP1+ cells. Subsequently, all BLIMP1+ cells became highly OCT4+ by day 4. By contrast, NANOG was expressed in ∼35% of BLIMP1+ cells on day 1, but it was generally absent in other cells in the embryoids (Figures 4D and S4A). Thereafter, NANOG was also rapidly upregulated in the majority of BLIMP1+ cells by day 2–4. The upregulation of key pluripotency genes, such as OCT4 and NANOG, is also reminiscent of their re-expression in mouse PGCs (Magnúsdóttir et al., 2013). Although NANOS3-mCherry expression was weakly detected in 24% of OCT4+ cells at day 2 (Figure S4C), it was detected in all OCT4+ cells on day 4, confirming their PGCLC identity.

PRDM14 is a key regulator of pluripotency in mouse and human ESCs (Chia et al., 2010; Grabole et al., 2013; Ma et al., 2011; Yamaji et al., 2013) and is a key regulator of mouse PGC specification (Yamaji et al., 2008). PRDM14 was generally downregulated in day 1–2 embryoids but was detectable in the nucleus of most BLIMP1+ cells by day 4 (Figure S4A). Notably, in a minority of BLIMP1/NANOG-positive hPGCLCs at day 8, PRDM14 was enriched in the cytoplasm (Figure S4A), which was the case in most of the gonadal hPGCs (Figure 2F). This is in marked contrast to the persistent nuclear PRDM14 expression in mouse PGCs (Grabole et al., 2013).

The SOX17/BLIMP1 double-positive cells were initially distributed randomly in day 1 embryoids (Figure 4A) but were then loosely organized in clusters and often a single cluster in day 2 embryoids. By day 4, generally one and occasionally two tight clusters of hPGCLCs were observed either at the core or periphery of each embryoid (Figure 4E). Cumulative observations suggest that SOX17/BLIMP1 might be among the key regulators of hPGCLC specification. Although OCT4 and NANOG were detected between days 1 and 2 in conjunction with NANOS3-mCherry and other PGC-specific genes from days 2–4, PRDM14 was upregulated more gradually in hPGCLCs and was subsequently detected in the cytoplasm of embryonic hPGCs. Following the early expression of SOX17 and BLIMP1 in hPGCLCs, these two transcription factors were also detected in embryonic hPGCs in vivo, as well as in TCam-2 (Figures 2E and 2H). These observations suggest that SOX17-BLIMP1 might be among the critical determinant of hPGC specification and maintenance.

### Role of BLIMP1 during hPGCLC Specification

**Figure 5:**
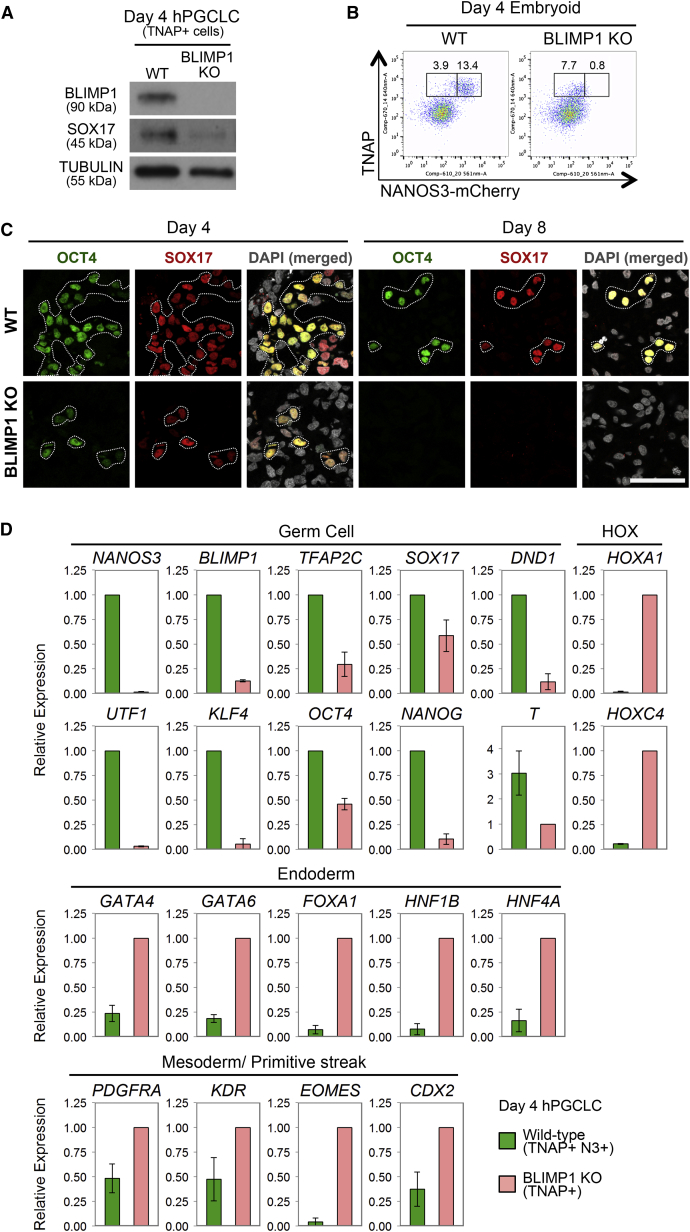
Role of BLIMP1 in hPGCLC Specification (A) Western blot analysis of BLIMP1 and SOX17 in TNAP-positive (TNAP+) cells sorted from wild-type (WT) and BLIMP1 knockout (BLIMP1 KO) day 4 embryoids after hPGCLC induction. TUBULIN was used as loading control. (B) FACS analysis of TNAP and NANOS3-mCherry on WT and BLIMP1 knockout (BLIMP1 KO) day 4 embryoids. (C) Immunofluorscence for OCT4 and SOX17 in cryosections of WT and BLIMP1 KO day 4 and 8 embryoids. OCT4-positive cells are highlighted. Scale bar, 50 μm. (D) Expression analysis by RT-qPCR for WT TNAP/NANOS3-mCherry double-positive cells (WT; TNAP+N3+) and BLIMP1 KO TNAP single-positive cells (BLIMP1 KO; TNAP+) sorted from day 4 embryoids. Relative expression levels are shown with normalization to *β−ACTIN*. Error bars indicate mean ± SD from two independent biological replicates.

**Figure S5:**
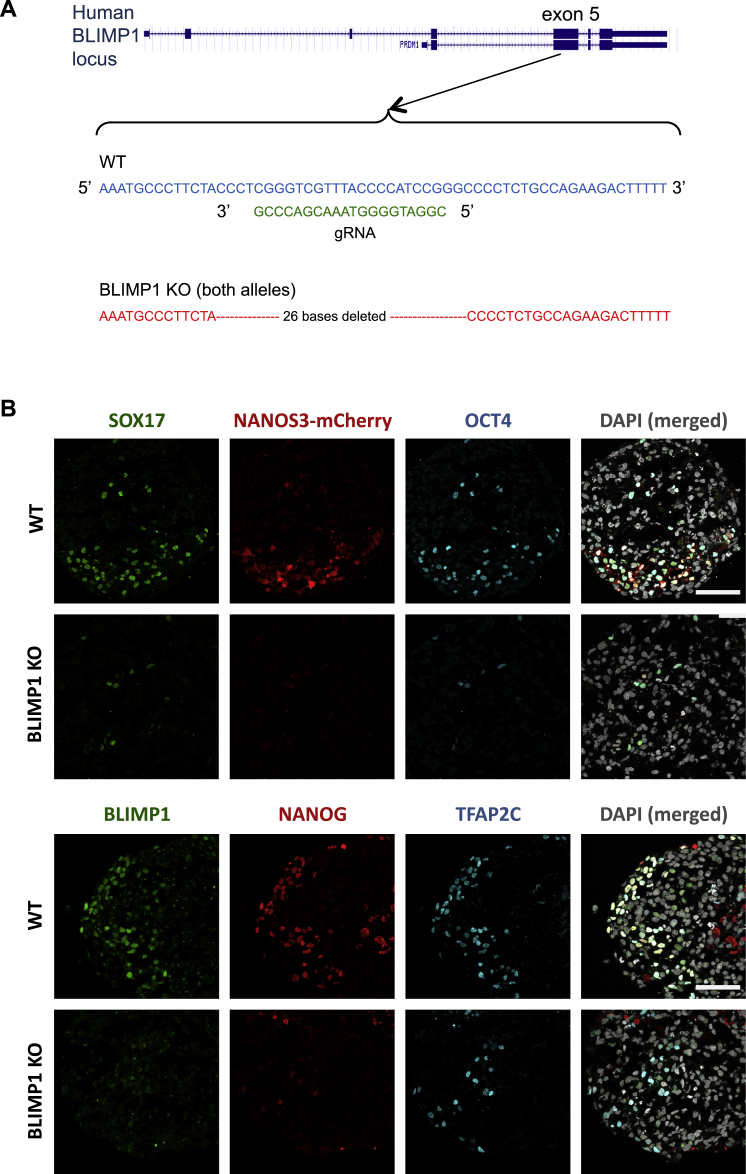
Generation of BLIMP1 KO in WIS2-NANOS3-mCherry hESC Line, Related to Figure 5 (A) Targeting strategy of BLIMP1 knockout in hESC with the designated guide RNA (gRNA) and the resulting deleted sequences. (B) Immunofluorescence of SOX17, NANOS3-mCherry and OCT4 (upper panel); and BLIMP1, NANOG and TFAP2C on wild-type (WT) and BLIMP1 KO day 4 embryoids. Scale bars = 70 μm.

BLIMP1 is the first and key regulator of mouse PGC, and loss of function abrogates PGC fate (Ohinata et al., 2005; Vincent et al., 2005). However, BLIMP1 expression is apparently downstream of SOX17 in hPGCLCs (Figures 4A and 4C). We examined its mechanistic role by generating *BLIMP1* knockout (KO) NANOS3-mCherry hESC line (Figure S5A). These cells showed loss of BLIMP1 by western blot (Figure 5A) and immunofluorescence (Figure S5B) on day 4 of hPGCLCs induction. Notably, there was also a loss of NANOS3-mCherry-positive cells, together with a significant reduction of NANOG, OCT4, and TFAP2C expression on day 4 (Figures 5C and S5B), indicating a failure of hPGCLC specification, and all of these cells disappeared by day 8 (Figure 5C). However, we detected ∼8% of TNAP-positive cells in day 4 embryoids (Figure 5B). This observation is highly reminiscent of the effects of *Blimp1* mutation on mouse PGC specification (Ohinata et al., 2005).

**Figure 6:**
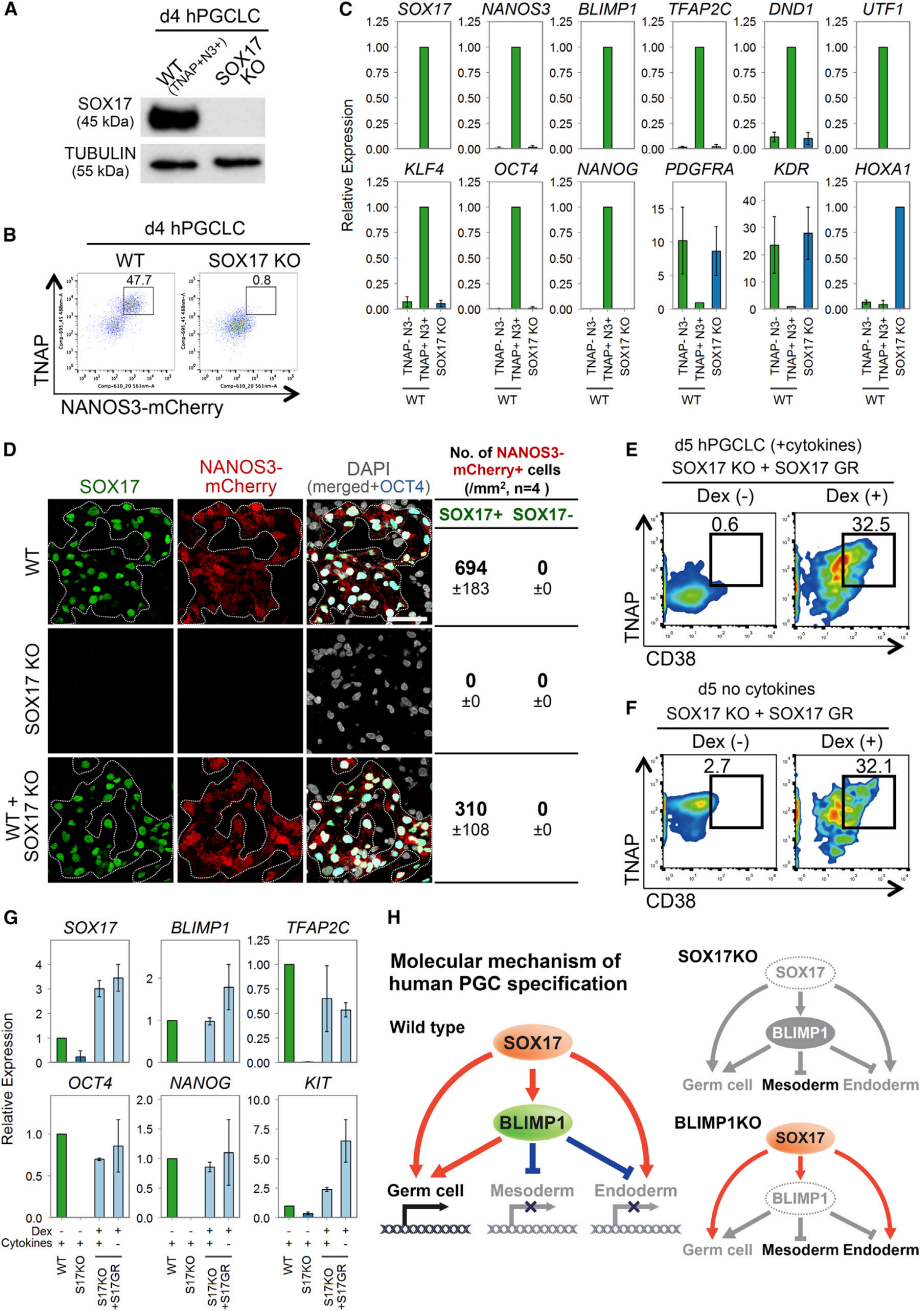
Role of SOX17 in hPGCLC Specification (A) Western blot analysis of SOX17 expression of WT day 4 TNAP/NANOS3-mCherry-positive hPGCLCs (WT, TNAP+N3+), and whole SOX17 knockout day 4 embryoids. TUBULIN was used as loading control. (B) FACS analysis of TNAP and NANOS3-mCherry on WT and SOX17 KO day 4 embryoids. (C) RT-qPCR analysis of TNAP/NANOS3-mCherry FACS-sorted WT double-negative (TNAP-N3-) or -positive (TNAP+N3+) cells sorted from day 4 embryoids and whole SOX17 KO embryoids (SOX17 KO). Relative expression levels are shown with normalization to *β−ACTIN*. Error bars indicate mean ± SD from two independent biological replicates. (D) Immunofluorescence of day 4 embryoids derived from WT, SOX17 knockout (SOX17 KO), and from 1 to 1 mixture of WT and SOX17 KO 4i hESCs. The number of NANOS3-mCherry+ cells with or without SOX17 expression is shown. Quantification was based on seven to nine confocal images from four independent embryoids of each condition. Scale bars, 50 μm. (E and F) FACS analysis of TNAP and CD38 on day 5 embryoids derived from SOX17 knockout 4i hESCs containing *SOX17* fusion construct with human glucocorticoid receptor ligand-binding domain (SOX17 KO+ SOX17 GR). Embryoids were derived in the presence (E) or absence (F) of cytokines with (Dex+) or without (Dex−) addition of dexamethasone. (G) RT-qPCR analysis of day 5 hPGCLC derived from WT and SOX17 KO (S17KO) and SOX17 KO + SOX17-GR (S17KO+S17GR) hESCs with (+) or without (−) dexamethasone (Dex) and in the presence (+) or absence (−) of cytokines. FACS-sorted NANOS3-mCherry/TNAP double-positive cells or whole embryoids (for S17KO) were used. Relative expression levels are shown with normalization to *GAPDH*. Error bars indicate mean ± SD from two biological replicates. (H) Model for establishment of hPGC transcription network by SOX17 and BLIMP1. SOX17 induces germ cell genes and, potentially, endoderm gene. Expression of BLIMP1, downstream of SOX17, suppresses endodermal genes, as well as mesodermal genes. As a result, the SOX17-BLIMP1 axis initiates hPGC program from competent cells upon induction by BMP signaling. The hPGC specification gene network is abrogated in the absence of SOX17 or BLIMP1.

We isolated and characterized the TNAP-positive cells by FACS and confirmed loss of *BLIMP1*, except for low expression of mutant transcripts (Figure 5D). These cells also showed loss of *NANOS3*, *UTF1*, and *KLF4* and reduced expression of *TFAP2C*, *DND1*, *OCT4*, *NANOG*, and *T* (Figures 5D and S5B). In addition, they showed prominent upregulation of mesodermal/primitive streak and *HOX* genes, as well as endodermal genes, including *GATA4*, *GATA6*, *FOXA1 HNF1β*, and *HNF4α* (Figure 5D). By contrast, endodermal genes were not upregulated in *Blimp1* mutant mouse PGCs (Kurimoto et al., 2008; Vincent et al., 2005). This suggests that BLIMP1 probably suppresses endoderm and other somatic genes, which might otherwise be induced by SOX17 and BMP signaling during hPGCLCs specification (Figure 6H). Loss of *BLIMP1* and *TFAP2C* also caused upregulation of *HOX* genes in TCam-2 (Weber et al., 2010). This suggests that one of the roles of BLIMP1 is to continually suppress the somatic program during human germline development.

### SOX17 Is the Key Regulator of hPGCLCs, which Acts Upstream of BLIMP1

**Figure S6:**
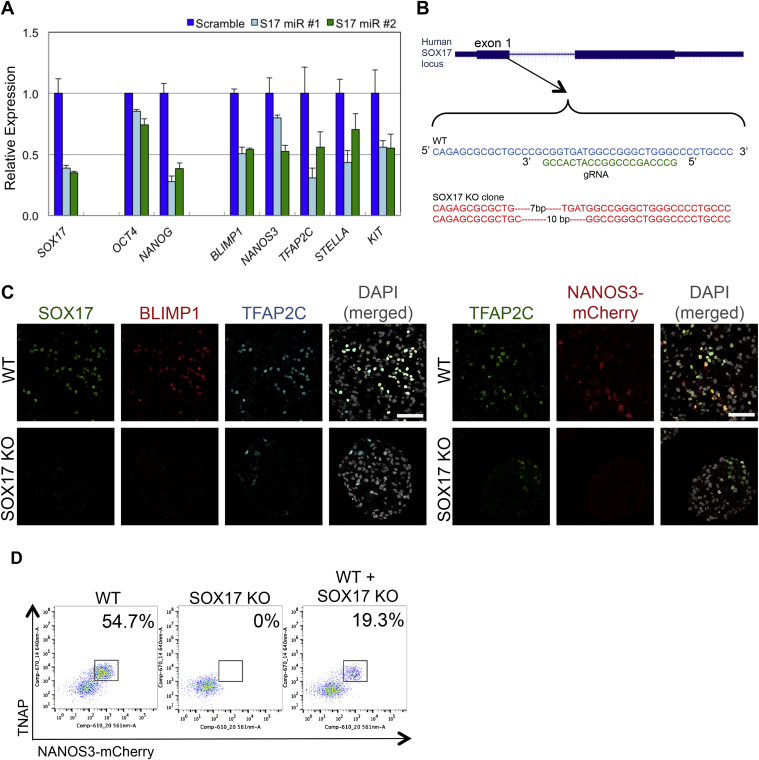
Generation of SOX17 KO in WIS2-NANOS3-mCherry hESC Line, Related to Figure 6 (A) Expression analysis by RT-qPCR of pluripotency and germ cell genes after knock-down of SOX17 in TCam-2. Two independent, miRs against SOX17 (S17 miR #1 and #2) were used for the knockdown with a scramble miR as control. Error bars are mean ± SD. Relative expression levels are shown with normalization to *GAPDH*. Representative data were shown from two independent biological replicates. (B) Targeting strategy of SOX17 knockout in hESC with the designated guide RNA (gRNA) and the resulting deleted sequences. (C) Immunofluorescence of SOX17, BLIMP1 and TFAP2C (left panel); and TFAP2C and NANOS3-mCherry on wild-type (WT) and SOX17 KO day 4 embryoids. Scale bars = 70 μm (D) FACS analysis of day 4 embryoids derived from wild-type (WT), SOX17 knockout (SOX17 KO) and 1 to 1 mixture of WT and SOX17 KO cells (WT + SOX17 KO).

Expression of SOX17 among T-positive cells prior to BLIMP1 apparently marks the onset of hPGCLC specification, which is a key difference between the specification of human and mouse germline fate (see Figure 4). Notably, SOX17 and BLIMP1 are also expressed in the authentic in vivo hPGCs and in TCam-2 (de Jong et al., 2008) (Figure 2). Knockdown of SOX17 in TCam-2, which exhibits key germ cell characteristics (Looijenga et al., 2014) (Figure 2), induced repression of the pluripotency genes *NANOG*, as well as of the PGC-genes *BLIMP1*, *NANOS3*, *TFAP2C*, *STELLA*, and *KIT* (Figure S6A). This suggests that SOX17 might be important for regulating the established germline gene expression network.

We addressed the role of SOX17 during hPGCLC specification by generating *SOX17* KO NANOS3-mCherry hESC line (Figure S6B) and validated absence of SOX17 expression in day 4 embryoids from mutant cells by western blot and immunofluorescence (Figures 6A and S6C). Notably, we did not detect any NANOS3-mCherry or TNAP-positive cells in the embryoids from SOX17 mutant cells (Figure 6B). Further, RT-qPCR analysis of day 4 SOX17 null embryoids showed absence of *NANOS3*, *TFAP2C*, *DND1*, *UTF1*, *KLF4*, *OCT4*, *NANOG*, and, importantly, *BLIMP1* (Figure 6C). Instead, there was upregulation of mesodermal genes *PDGFRA*, *KDR*, and *HOXA1* (Figure 6C). Although a few TFAP2C-positive cells were detected on day 4, they were BLIMP1 negative and most likely belong to other lineages (Figure S6C).

To determine whether SOX17 acts cell autonomously, we mixed wild-type NANOS3-mCherry hESCs with the *SOX17* null hESCs in 1:1 ratio during induction of hPGCLCs by cytokines. All NANOS3-mCherry positive cells detected by immunofluorescence on day 4 were SOX17 positive (Figure 6D), indicating that SOX17 null hESCs did not undergo hPGCLC specification even in the presence of wild-type cells. The overall number of NANOS3-mCherry-positive cells in the embryoid with mixed cells was about half of that in the control consisting of wild-type cells only (Figure S6D), suggesting that SOX17 null cells did not affect PGCLC induction from wild-type cells. Thus, SOX17 null cells have intrinsic defect for hPGCLC specification.

To determine the competency of the *SOX17* null hESCs, we transfected an inducible SOX17 fusion construct with human glucocorticoid receptor ligand-binding domain (GR) into the SOX17 null hESCs. This would allow dexamethasone (Dex) to activate the SOX17-GR and induce translocation of SOX17 fusion protein from the cytoplasm into the nucleus (Brocard et al., 1998). After 5 days of induction with cytokines and Dex in the SOX17 null SOX17-GR hESCs, expression of germ cell genes *BLIMP1*, *TFAP2C*, *OCT4*, *NANOG*, and *KIT* and the TNAP/CD38-positive population was restored (Figures 6E and 6G). This demonstrates that SOX17 null hESCs maintain competency for hPGCLC specification. Strikingly, activation of SOX17 alone in the absence of cytokines was sufficient to induce germ cell genes and TNAP/CD38-positive cells from 4i hESCs (Figures 6F and 6G). Taken together, SOX17 is indispensable and sufficient for hPGCLC gene induction from competent hESCs, and it acts upstream of BLIMP1 and other genes to initiate the human germ cell transcriptional network (Figure 6H). Interestingly, loss of SOX17 in TCam-2 also causes a repression of germ-cell- and pluripotency-associated genes (Figure S6A). This suggests that SOX17 might also be important for the maintenance of the germ cell state because it is also highly expressed in embryonic hPGCs.

### Specification of hPGCLCs from Germ-Cell-Competent hESC/hiPSCs

**Figure 7:**
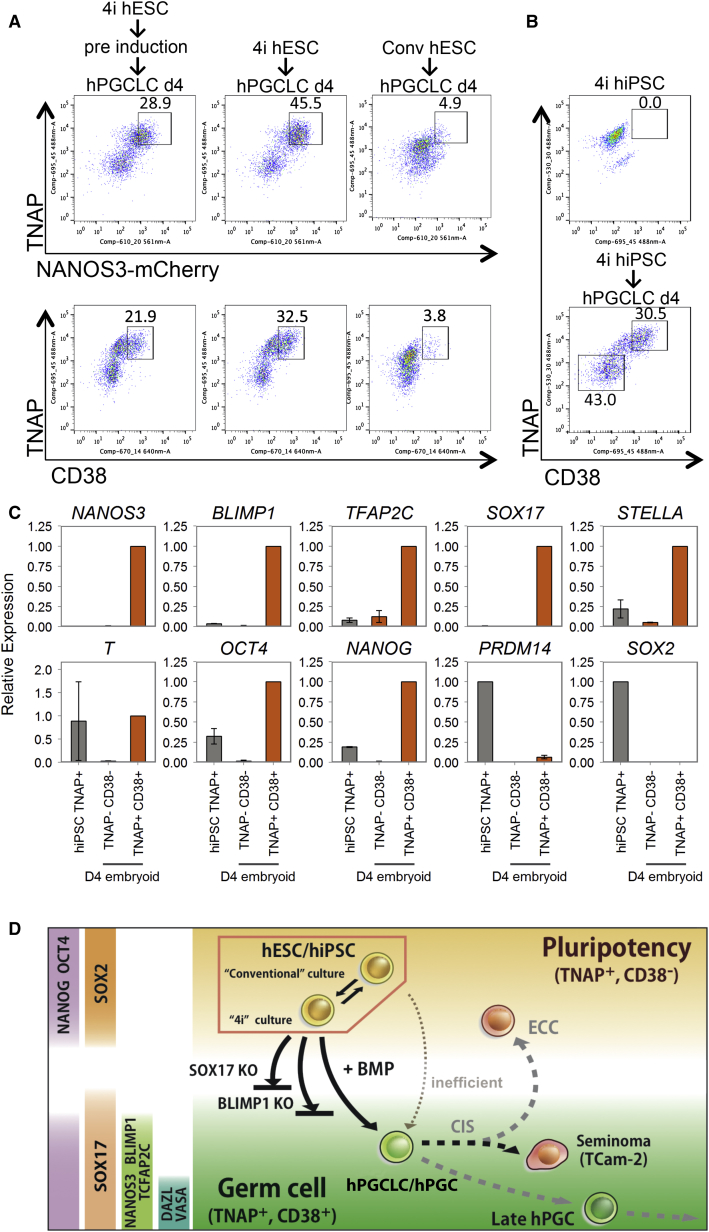
Induction and Isolation of hPGCLCs from Competent hiPSCs/hESCs (A) FACS analysis of TNAP and NANOS3-mCherry (top) and TNAP and CD38 (bottom) on day 4 embryoids induced from 4i hESCs after preinduction (left), directly without preinduction (middle) or from conventional hESCs (right, Conv hESC). (B) FACS analysis of TNAP and CD38 in 4i hiPSCs (top) and day 4 embryoids derived from 4i hiPSCs after direct induction (bottom). (C) Expression analysis by RT-qPCR on TNAP-positive hiPSCs (iPSC TNAP+), TNAP/CD38 double-negative (TNAP−CD38−) population and TNAP/CD38 double-positive population (TNAP+CD38+) on day 4 after hPGCLC induction. Relative expression levels are shown with normalization to *β−ACTIN*. Error bars indicate mean ± SD from two independent biological replicates. (D) Overview of human germline development. hESCs in 4i reversibly attains competence for germ cell fate. Exposure of 4i cells to cytokines containing BMPs results in strong induction of hPGCLCs following expression of SOX17-BLIMP1, which are among the key regulators of germ cell fate. SOX17 and BLIMP1 are detected in in vivo gonadal hPGC and TCam-2 seminoma, indicating a likely progression of early human germ cell lineage. CD38, a cell-surface glycoprotein, is shared by all cells with germ cell characteristics, but not by hESC. Loss of *SOX17* or *BLIMP1* abrogates hPGCLC specification.

Because gene expression of hESCs in 4i medium resembles that of hESC after preinduction for 2 days in bFGF/TGFβ (Figures 2A, 2B, and S2A), we decided to investigate hPGCLC induction directly in hESCs maintained in 4i medium (Figure 1A). Indeed, hPGCLCs could be induced directly from 4i hESCs with apparent enhanced response resulting in ∼46% hPGCLCs (Figure 7A). These hPGCLCs showed a slightly higher intensity of NANOS3/TNAP by FACS, and a greater proportion of them were CD38 positive (Figure 7A). Notably, cells maintained for more than 2 weeks in the conventional hESC medium, regardless of whether they were initially maintained in 4i medium, showed a significantly lower numbers of hPGCLCs (∼5%) with a reduced intensity of NANOS3-mCherry/TNAP and CD38 expression (Figure 7A). This demonstrates that hESCs in 4i medium are highly competent for the hPGCLC fate. Importantly, the competent state is conferred reversibly because it is gained and lost in 4i and conventional culture conditions, respectively.

**Figure S7:**
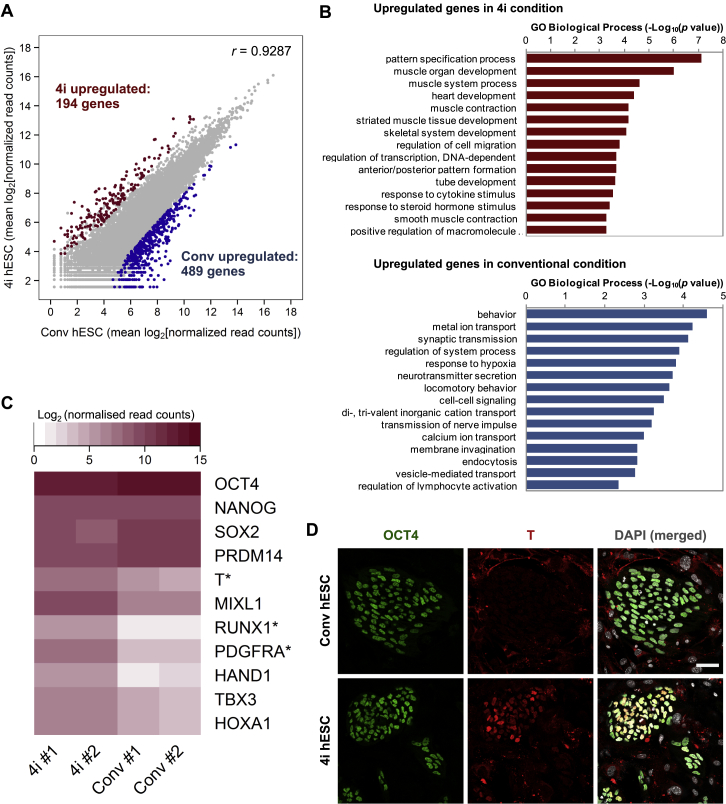
Comparison of hESC Transcriptomes under Conventional or 4i Culture Conditions, Related to Figure 7 and Table S2 (A) Scatter plot showing global gene expression levels (mean log_2_(normalized read counts) of two replicates) between WIS2 hESC cultured under 4i and conventional condition. Each dot represents one gene. Differentially expressed genes (log_2_(fold change) > 2 and adjusted *p* value < 0.05) were presented as red dots (upregulated in 4i condition) or blue dots (upregulated in conventional condition). (B) Gene ontology (GO) term enrichment analysis of upregulated genes in 4i WIS2 hESC (upper panel) and conventional WIS2 hESC (lower panel). Top 15 GO biological process terms that were enriched in each condition were shown (DAVID GOTERM_BP_FAT with gene count > = 5 followed by GO Trimming to reduce term redundancy). (C) Heat map showing expression of representative pluripotency and mesodermal genes expression in WIS2 hESCs cultured under 4i or conventional conditions (Conv). RNASeq data from two biological replicates were shown (#1 and #2). Asterisk indicates differential expression with statistical significance (log_2_(fold change) > 2 and adjusted *p* value < 0.05). (D) Immunofluorescence analysis of T and OCT4 on WIS2 hESCs cultured under conventional (Conv hESC) and 4i condition (4i hESC).

Global gene expression analysis indicated overall similarities between hESCs in the conventional medium versus those in “4i” medium (r = 0.923) but with notable differences (Figure S7A). Although these cells showed similar expression levels of core pluripotency factors *OCT4*, *NANOG*, and *SOX2*, 4i hESCs had higher expression of mesoderm and gastrulation genes, including *T*, *RUNX1*, and *PDGFRA* (Figures S7B and S7C and Table S2). Furthermore, OCT4-positive cells in 4i hESCs had varying levels of T protein, possibly due to inhibition of GSK3β (Chen et al., 2013), which is not the case in hESC cultured in conventional condition (Figure S7D). These differences might be relevant for the mechanism of competence of ESCs for PGCLC, which merits further investigation.

We also asked whether hiPSCs could be used to generate and isolate hPGCLCs using the combination of surface markers CD38 with TNAP (Figures 2C and 3A–3D). Using FX71.1 hiPSCs (see Experimental Procedures) maintained in 4i medium for >2 weeks that lack CD38 expression, we detected∼31% of TNAP/CD38 double-positive cells after 4 days in response to cytokines (Figure 7B). TNAP/CD38 double-positive hPGCLCs showed expression of *NANOS3*, *BLIMP1*, *TFAP2C*, *SOX17*, *STELLA*, *T*, *OCT4*, *NANOG*, and *PRDM14*, but not of *SOX2* (Figure 7C). Similar results were obtained with another hiPSC line (C1, Gafni et al., 2013). Thus, hPGCLC specification could be induced efficiently and directly in hiPSCs that are maintained in the 4i medium, which could be used for disease modeling using patient-derived iPSCs.

## Discussion

Specification of hPGCLCs from germ cell competent hESC/hiPSC provides a unique mechanistic view of the establishment of the human germline (Figure 7D). Notably, SOX17 is the key regulator of hPGCLC specification, whereas BLIMP1 represses endodermal and other somatic genes during hPGCLC specification. This was unexpected because the primary role of SOX17 is in the endoderm (D'Amour et al., 2005; Kanai-Azuma et al., 2002) and because *Sox17* has no detectable role in the specification of mouse PGCs (Hara et al., 2009; Kanai-Azuma et al., 2002). A comparison among hPGCLCs, embryonic hPGCs, and TCam-2 seminoma (Looijenga et al., 2014; Schafer et al., 2011) also establishes the likely progression of the early human germline (Figure 2B).

During hPGCLC specification from hESCs, SOX17 was first detected in a few scattered cells in day 1 embryoids, which showed expression of T. The nascent hPGCLCs subsequently form a few or a single cluster in day 4–8 embryoids. SOX17 is indeed essential for hPGCLC specification, and this gene alone is sufficient to induce germ cell genes in the *SOX17* mutant cells, with or without cytokines from 4i hESCs. SOX17 acts cell autonomously, and the presence of mutant cells in embryoids had no effect on hPGCLC specification from wild-type cells. It will be of interest to see how SOX17, with or without BLIMP1, determines cell fates between germ cell, hematopoietic, and endodermal lineages (Nakajima-Takagi et al., 2013; Clarke et al., 2013).

Expression of BLIMP1 is intimately associated with SOX17 during hPGCLC specification. BLIMP1 represses somatic genes, including mesendodermal genes, which might allow SOX17 to function as the regulator of hPGCLCs specification. A mutation in BLIMP1 abrogates hPGCLC specification but without completely abolishing SOX17 expression. However, TNAP-positive cells were detected, in which PGC-specific genes were repressed but some endodermal and other somatic genes were upregulated. This suggests that BLIMP1 might repress them during hPGCLC specification, but not excluding its wider role in hPGCLC specification in conjunction with SOX17. In mice, BLIMP1 also represses somatic genes in PGCs (Ohinata et al., 2005; Vincent et al., 2005), but it is also a key determinant of PGC specification, together with PRDM14 and TFAP2C (Magnúsdóttir et al., 2013).

Although PRDM14 is critical for mouse PGC specification, its expression during hPGCLC specification is delayed and significantly diminished in hPGCs and is very low in TCam-2 compared to hESCs. PRDM14 is crucial for maintaining pluripotency in human and mouse ESCs, although different signaling molecules regulate its expression, and the genomic targets in ESCs also differ in the two species (Chia et al., 2010; Grabole et al., 2013; Ma et al., 2011; Yamaji et al., 2013). The rapid downregulation and delayed re-expression of PRDM14 at the onset of hPGCLC induction (Figures 2F and S4A) may allow exit of pluripotency from 4i hESC en route to germ cell differentiation. Interestingly, the human and mouse PRDM14 proteins have diverged, which might result in functional differences. There is expression of SOX2 in mouse PGCs, which is apparently regulated by PRDM14 (Grabole et al., 2013), whereas SOX2 is repressed in human hPGCLCs/hPGCs. BLIMP1 also apparently represses SOX2 during spontaneous differentiation of hPGCLCs from hESCs (Lin et al., 2014). By contrast, KLF4 is expressed in hPGCLCs /hPGCs (Figure 2C), but not in mouse PGCs (Kurimoto et al., 2008). The precise significance of the repression and expression of pluripotency genes, including NANOG, remains to be elucidated.

Germ cell neoplasia or carcinoma in situ (CIS) (Skakkebaek, 1972) can generate embryonal carcinoma cells that resemble hESCs or seminomas such as TCam-2 that inherit key characteristics of germ cells (Looijenga et al., 2014; Schafer et al., 2011). TCam-2 expresses *SOX17*, *BLIMP1*, *TFAP2C*, *KIT*, and *DND1* with low levels of *SOX2* and *PRDM14*. Knockdown of SOX17 in TCam-2 induces repression of germ cell and pluripotency genes (Figure S6A), whereas knockdown of BLIMP1 and TFAP2C induced upregulation of somatic genes (Weber et al., 2010). These observations suggest that SOX17 and BLIMP1 might also be important for the maintenance of the early human germline. We found that CD38 is a marker of all human germline-related cells, including seminomas. Distinction between seminoma and embryonal carcinoma could therefore be made by the expression of SOX17/CD38 and SOX2/CD30, respectively (de Jong et al., 2008). Furthermore, CD38/TNAP are reliable markers for the isolation of hPGCLCs derived from hESC/hiPSC without any reporters.

The hPGCLCs also showed early signs of DNA demethylation, which is consistent with the germline-specific epigenetic program. The striking upregulation of 5hmC concomitantly with TET1 suggests that, similar to mouse, conversion of 5mC to 5hmC may contribute to DNA demethylation in hPGC (Hackett et al., 2013). Furthermore, repression of UHRF1 and DNMT3A in hPGCLCs would promote DNA-replication-coupled loss of 5mC. Indeed, there was a small but significant decline in 5mC in hPGCLCs, a trend that could lead to a significant loss of 5mC with further proliferation of hPGCLCs. Furthermore, we detected upregulation and translocation of PRMT5 to the nucleus in hPGCLC, which occurs with the onset of global DNA demethylation to repress transposable elements (Kim et al., 2014). Detailed analysis of the transcriptome and epigenome, together with the targets of SOX17 in hPGCLCs/hPGCs, should provide insights on the mechanism of how the epigenome is reset in the early human germline and potentially on the inheritance and consequences of transgenerational epigenetic inheritance (Heard and Martienssen, 2014).

This study shows that changes in pluripotent cell states can be induced by environmental factors with respect to gain and loss of competence for germ cell fate in hESCs in the 4i culture (Gafni et al., 2013). This competence for hPGCLCs is reversibly maintained and progressively lost in conventional culture conditions. Notably, hESCs in 4i medium show a slight upregulation of T together with HAND1 compared to conventional hESCs (Figure S7), with putative posterior primitive streak-like feature (Mendjan et al., 2014). This might explain why hESC in 4i are highly competent for hPGCLC fate. Because MAPK inhibitors may also alter the epigenetic state of pluripotent cells (Gafni et al., 2013), the precise molecular basis for competence for PGC fate remains to be elucidated in both mouse and human. Nonetheless, hESC/hiPSC can reversibly gain competence for hPGCLC specification in 4i medium, which provides a model for advances in human germ cell biology.

Mouse is the primary model organism for early mammalian development, pluripotency, and the regulation of cell fates. Postimplantation rodent embryos develop as egg cylinders with an overlying extraembryonic ectoderm, which is the source of signals, including BMP4, whereas postimplantation epiblast embryonic disc in humans is typical of many mammalian species (Barrios et al., 2013; de Fellici, 2013; Irie et al., 2014). These differences may affect the source, duration, and the nature of signaling molecules that regulate competence for cell fates in vivo. The evolutionary divergence in the pluripotent states in mouse and human might also result in differences in the mechanism of germline specification and, potentially, other cell fate decisions. If so, mechanisms of early cell fate decisions in mice cannot be safely or wholly extrapolated to specification events during early human development.

## Experimental Procedures

### hESC/iPSC Culture and hPGCLC Differentiation

4i hESCs (WIS2: 46XY; WIBR3: 46XX; LIS1, 46XY) and iPSCs (FX71.1; a fragile X male patient-derived iPSC line, C1 female iPSC line) were grown in conditions adapted and modified from previously described WIS-NHSM conditions (Gafni et al., 2013). 4i cells were grown on irradiated mouse embryonic fibroblasts (MEFs) (GlobalStem) in knockout DMEM supplemented with 20% knockout serum replacement (KSR), 2 mM L-glutamine, 0.1 mM nonessential amino acids, 0.1 mM 2-mercaptoethanol (all GIBCO), 20 ng/ml human LIF (Stem Cell Institute [SCI]), 8 ng/ml bFGF (SCI), 1 ng/ml TGF-β1 (Peprotech), 3 μM CHIR99021 (Miltenyi Biotec), 1 μM PD0325901 (Miltenyi Biotec), 5 μM SB203580 (TOCRIS bioscience), and 5 μM SP600125 (TOCRIS bioscience). Cells were passaged every 3 to 5 days using TrypLE Express (GIBCO). 10 μM of ROCK inhibitor (Y-27632, TOCRIS bioscience) was used for 24 hr after the passage.

To preinduce, 4i hESCs were dissociated with TrypLE Express and filtered with 50 μm cell filter (PERTEC), and 4 × 10^5^ cells/ 12-well were plated on vitronectin/gelatin-coated plates (Gafni et al., 2013) in N2B27 medium (Ying et al., 2008) with 1% KSR, 10 ng/ml bFGF (SCI), 1 ng/ml TGF-β1 (Peprotech), or 20 ng/ml Activin A (SCI) and 10 μM ROCK inhibitor. Medium was changed on day 1. After 2 days of preinduction, the cells are dissociated with TrypLE and plated to ultra-low cell attachment U-bottom 96-well plates (Corning, 7007) at a density of 2,000–4,000 cells/well in 200 μl PGCLC medium. PGCLC medium is composed of Glasgow’s MEM (GMEM, GIBCO), 15% KSR, 0.1 mM nonessential amino acids, 0.1 mM 2-mercaptoethanol, 100 U/ml Penicillin-0.1 mg/ml Streptomycin, 2 mM L-Glutamine, 1 mM Sodium pyruvate, and the following cytokines: 500 ng/ml BMP4 (R&D Systems) or BMP2 (SCI), 1 μg/ml human LIF (SCI), 100 ng/ml SCF (R&D Systems), 50 ng/ml EGF (R&D Systems), and 10 μM ROCK inhibitor.

Conventional hESCs/hiPSCs were maintained on irradiated MEFs (GlobalStem) in DMEM/F12+GlutaMAX supplemented with 20% KSR, 0.1 mM nonessential amino acids, 0.1 mM 2-mercaptoethanol (all GIBCO), and 10–20 ng/ml of bFGF (SCI). Media were replaced every day. Cells were passaged every 4 to 6 days using 1 mg/ml of Dispase (GIBCO), and 10 μM ROCK inhibitor (Y-27632, TOCRIS bioscience) was added for 24 hr after the passage.

Extended Experimental Procedures **Genomic Modifications in hESCs**

In order to introduce 2A-mCherry sequence immediately downstream and in frame with the coding sequence of NANOS3, a donor construct was produced as depicted in Figure S1A. Homology arms were amplified using the primers: 5′ homology forward with Not I restriction enzyme site: at gcggccgc gtgcctgtagtcccagctacttgggag, 5′ homology reverse with SalI restriction enzyme site: acc gtcgac tctagagtcgcgaccgctgta ggtggacatggagggagagcagg, 3′ homology forward with HpaI restriction enzyme site: at gttaac gaggctgcctacacctgggca and 3′ homology reverse with HpaI restriction enzyme site: cg gttaac aagatctggaggtggaggaggcag. TALEN expressing constructs were generated using GoldenGate TALEN kit 2.0 (Addgene cat#1000000024). TALEN’s repeats sequences targeting human NANOS3 stop codon were: forward HD HD NG NH HD NG HD NG HD HD HD NG HD HD NI NG, and reverse NG NH HD HD HD NI HD HD NG NH NG NI NH NH HD NI. WIS2 and LIS1 male hESC and WIBR3 female hESC lines were electroporated with a pair of TALEN coding plasmids and donor construct. After selection with G418 (150 μg/ml) and ganciclovir (2 μM), genomic DNA was extracted from 96 clones for each cell line. Targeting efficiency was about 50%–60% in all the experiments as revealed by PCR and Southern Blot analysis. Southern blot with internal anti-mCherry probe did not show non-specific insertions in 80% of correctly targeted clones. In order to delete PGK-Neo cassette, correctly targeted clones were transfected with flippase expressing plasmid and subcloned. Neo cassette excision was confirmed by PCR. Karyotyping analysis of correctly targeted clones was performed by G-Banding on ASI platform, and confirmed normal karyotype in all clones used.

In order to knock out BLIMP1 and SOX17 genes, oligos encoding gRNAs targeting these genes were inserted into px330 vector (Cong et al., 2013). Unique gRNA sequences were chosen with the help of Zhang Lab website http://www.genome-engineering.org/crispr/ (Figures S5A and S6B). 100 μg of resulting construct and 10 μg of GFP expressing vector were electroporated into WIS2-NANOS3-mCherry hESCs. 3 or 4 days later, GFP expressing cells were sorted by FACS and seeded at low density. 9 days after seeding 88 colonies were picked for each experiment and genomic DNA was extracted. DNA was analyzed by High Resolution Melt assay (HRM) using MeltDoctor reagent (Life Technologies) and the clones that showed reduced Tm for both alleles compared to wild-type controls were expanded. In these selected clones targeted locus was amplified and sequenced. Primers for HRM are; BLIMP1: CGATGACTTTAGAAGACGTGGAGCC; CGTAGGCCAGGGAAGCTTTCAA, SOX17: GCCAGTGACGACCAGAGCCAG; TCACCTTCATGTCCCCGATGG. Electroporation protocol used in all the experiments was described previously (Gafni et al., 2013). Gene targeting plasmids are available through Addgene.

SOX17 KO + SOX17-GR hESCs were generated by transduction of PiggyBac based vector which enables to express fusion protein of SOX17 with GR (human glucocorticoid receptor ligand-binding domain) under the control of CAG promoter into SOX17 KO hESCs. This system allows inducing the nuclear translocation of SOX17 by addition of 1 μg/ml dexamethasone (Dex).

**Fluorescence-Activated Cell Sorting**

Day 1-8 embryoids were washed in PBS and dissociated with TrypLE Express for 8-15 min at 37°C. Day 6 or later embryoids were pretreated with 0.38 mg/ml EGTA and 10 mg/ml PVA in PB1 on ice for 10 min before dissociation. Human embryonic genital ridges were dissected out from surrounding somatic tissues in PBS and dissociated with TrypLE at 37°C for 30 min. Dissociated cells were resuspended in FACS solution consisted of 3% (v/v) fetal bovine serum (FBS) in PBS. Samples were incubated with anti-alkaline phosphatase (TNAP) antibody (BD PharMingen), anti-KIT antibody (Molecular Probes) and/or anti-CD38 antibody (BioLegend) conjugated with PerCP-Cy5.5, Alexa Fluor 488 or Alexa Fluor 647. After washing with PBS, the cells were treated with 1 μg/ml DAPI and were analyzed by BD LSRFortessa (BD Biosciences) or sorted by MoFlo (Beckman Coulter) or S3 Cell Sorter (Bio-Rad).

**Real-Time Quantitative RT-PCR**

Total RNA was extracted using RNeasy Kit (QIAGEN) or PicoPure RNA Isolation Kit (Applied Biosystems) and cDNA was synthesized using QuantiTect Reverse Transcription Kit (QIAGEN). qRT-PCR were performed and analyzed as described previously (Grabole et al., 2013) and the primers used are shown in Table S3. Values normalized to *β−ACTIN* or *GAPDH* and relative to control samples are shown. Error bars are mean ± SD from two or three independent experiments.

**Collection of Human Embryonic Tissues**

Human embryonic tissues were used under permission from NHS Research Ethical Committee, UK (REC Number: 96/085). Human embryonic samples were collected following medical or surgical termination of pregnancy carried out at Addenbrooke’s Hospital, Cambridge, UK with full consent from patients. Crown-rump length, anatomical features, including limb and digit development, was used to determine developmental stage of human fetuses with reference to Carnegie staging (CS). Genders of embryos were determined with by sex determination PCR of somatic tissues as described (Bryja and Konecny, 2003).

**RNA-Seq Samples Collection and Library Preparation**

For RNA-Seq data in Figure 2, WIS2 hESCs in 4i medium were sorted directly into extraction buffer (PicoPure RNA Isolation Kit (Applied Biosystems)) by FACS using Alexa Fluor 647-conjugated mouse anti-alkaline phosphatase antibody (BD PharMingen, 561500). For hPGCLCs from day 4 embryoids, TNAP and NANOS3-mCherry-double positive cells were sorted. Embryonic hPGCs, which were double positive for TNAP and KIT, were isolated from CS18 male genital ridge using Alexa Fluor 488-conjugated mouse anti-alkaline phosphatase antibody (BD PharMingen, 561495) and APC-conjugated mouse anti-KIT antibody (Molecular Probes, CD11705). The double negative population was collected as gonadal somatic cells (soma). Gonadal hPGCs were isolated to > 95% purity as determined by alkaline phosphatase staining using Leukocyte Alkaline Phosphatase Kit (Sigma). Pre-induced cells and TCam-2 were unsorted. Total RNA was extracted with the PicoPure RNA Isolation Kit. Total RNA (0.5-2 ng) was reverse transcribed and amplified into cDNA using Ovation RNA-Seq System V2 (Nugen) according to manufacturer’s instructions. Amplified cDNA was fragmented (peak at ∼250 bp) by Covaris S220 Focused-ultrasonicators. Subsequently, RNA-Seq library was generated with 500 ng of fragmented cDNA using Ovation Ultralow DR Multiplex System (Nugen). Library was quantified by KAPA Library Quantification Kit (Kapa Biosystems) using QuantStudio 6 Flex Real-Time PCR System (Applied Biosystems). Libraries were subjected to single-end 50 bp sequencing on HiSeq 2000 sequencing system (Illumina). Every 4 indexed libraries were multiplexed to one lane of a flowcell, resulting in > 40 million single end reads per sample. For WIS2 4i and conventional hESCs in Figure S7, total RNA was extracted using DirectZol RNA mini-prep (Zymo research, R2052). RNA integrity was evaluated on Tapestation (Agilent). Libraries were prepared by the INCPM unit in the Weizmann Institute of Science according to Illumina’s instructions accompanying the TruSeq RNA Sample Preparation Kit v2 (RS-122-2001). Sequencing was carried out on Illumina HiSeq2500 according to the manufacturer’s instructions, using 10 pM template per sample for cluster generation, and sequencing kit V2 (Illumina), resulting in ∼40 million paired-end reads per sample.

**Bioinformatic Analysis of RNA-Seq**

Before mapping, reads were quality-trimmed (Q > 20) and adaptor sequence was removed using TrimGalore (www.bioinformatics.babraham.ac.uk/projects/trim_galore). Reads were mapped to the human reference genome (GRCh37/hg19) by Tophat version 2.0.10 (ccb.jhu.edu/software/tophat). Transcripts with less than 100 read counts summed over all cell types were removed, and the R Bioconductor/DESeq package (bioconductor.org/packages/release/bioc/html/DESeq.html) was used to normalize counts per RefSeq transcripts to evaluate differential expression. Before clustering and principal component analysis, the transcripts with the 10% lowest average expression were removed, and the gene expression data matrix was centered and scaled. Time points were clustered hierarchically using Ward’s method and the Pearson correlation coefficient (1-c) as a distance measure. Principal component analysis was performed by a singular value decomposition (SVD) of the center-scaled gene expression data matrix. For comparison between hPGCLC, fetal hPGC and TCam-2, highly upregulated genes of each sample, with mean log_2_(normalized read counts) > 1, log_2_(fold change) > 3 and adjusted *p* value < 0.05 against soma, were selected and plotted as Venn diagram using BioVenn (Hulsen et al., 2008). Gene ontology analysis was performed by DAVID (Huang et al., 2009) followed by GO trimming (Jantzen et al., 2011).

**Immunofluorescence**

Embryoids containing hPGCLC or human embryonic tissues were fixed in 4% paraformaldehyde in PBS at 4°C for 1 hr. Samples were then washed twice in PBS and incubated sequentially with 10% and 20% sucrose each for 1 hr at 4°C. Subsequently, samples were embedded in OCT embedding matrix (CellPath), frozen by dry ice and stored at −80°C. Samples were prepared as 8 μm cryosections on Superfrost Plus Micro slides (VWR) by a cryostat (Leica, 3050S). Before immunofluorescence, slides with cryosections were air-dried for 1 hr. For immunofluorescence on 4i hESC and TCam-2, cells were grown on 8-well μ-Slide (ibidi, 80826). Glass slides with cryosections or cells cultured on μ-Slides were washed in PBS for 3 times with 10 min each. For 5mC and 5hmC staining, cryosections were subjected to heat-induced epitope retrieval in TE buffer (pH8) at around 95°C by a microwave oven. After retrieval, slides were cooled down to room temperature and washed in PBS for three times with 5 min each. Specimens were permeabilized in PBST (PBS with 0.1% Triton X-100) for 30 min, and then incubated with blocking solution (5% (v/v) normal donkey serum, 1% (w/v) bovine serum albumin in PBST) for 1 hr. Slides were then incubated with primary antibodies diluted in blocking solution at 4°C overnight. Subsequently, slides were washed in PBST for three times with 10 min each. Alexa Fluorophore (488, 568 and/or 647)-conjugated secondary antibodies derived from donkey (Molecular Probes) host species of the primary antibodies were diluted in PBST (1:300) with 1 μg/ml DAPI and incubated with the slides in dark at room temperature for 1 hr. Subsequently, slides were washed in PBS for three times for 10 min each and mounted with Prolong Gold Antifade Reagent (Molecular Probes). Images were taken using Leica TCS SP8 or SP5 confocal microscope. For consistency, immunostainings were performed simultaneously and imaged with fixed laser power settings. Primary antibodies used were: Rabbit anti-5hmC (1:500, Active Motif, 39769), Mouse anti-5mC (1:150, abcam, ab10805), Rabbit anti-TFAP2C (1:200, Santa Cruz, sc-8977), Rat anti-BLIMP1 (1:100, eBioscience, 14-5963), Rabbit anti-DAZL (1:250, abcam, ab34139), Rabbit anti-DNMT3A (1:100, Santa Cruz, sc-20703), Rabbit anti-Ki67 (1:100, abcam, ab16667), Goat anti-NANOG (1:100, R&D Systems, AF1997), Mouse anti-OCT4 (1:500, BD Biosciences, 611203), Goat anti-OCT4 (1:500, Santa Cruz, sc-8629), Rabbit anti-PRDM14 (1:100, Millipore, AB4350), Rabbit anti-PRMT5 (1:250, Millipore, 07-405), Rat anti-RFP (1:1000, Chromotek, 5F8), Goat anti-SOX17 (1:500, R&D Systems, AF1924), Goat anti-SOX2 (1:200, Santa Cruz, sc-17320), Goat anti-T (1:500, R&D Systems, AF2085), Mouse anti-TET1 (1:250, Genetex, GT1462), Mouse anti-UHRF1 (1:200, Active Motif, 61342), Goat anti-VASA (1:500, Active Motif, 61342).

**Image Analysis**

Analyses and quantification were performed with Volocity 3D Image Analysis Software (PerkinElmer). To quantify the fluorescence intensity of epigenetic modifications or modifiers in confocal images of embryoid cryosections (Figure 3G), a custom workflow was designed in Volocity. Briefly, each individual nucleus was selected based on DAPI signal. Nuclei which overlap with OCT4, BLIMP1 or TFAP2C signals were defined as PGCLCs, while the rest were defined as neighboring somatic cells. The fluorescence intensities for the epigenetic modifications or modifiers of interest were then measured in the two populations of nuclei and the distribution was plotted as boxplots. For UHRF1, only nuclei which were positive for KI-67 were included for quantification. Quantification was based on confocal images of at least 3 independent embryoids. To quantify the proportion of SOX17-positive cells that were positive for BLIMP1 or TFAP2C (Figures 4A and 4C), the number of SOX17 expressing cells were first counted manually. Among the SOX17-positive cells, the number of cells which express BLIMP1 or TFAP2C were quantified. The proportion was then obtained by dividing BLIMP1 (or TFAP2C) and SOX17 double positive cells over the total number of SOX17-positive cells. Proportions of BLIMP1-positive cells co-expressing TFAP2C, NANOG or OCT4 (Figure 4D) were quantified in similar manner. Quantification of each immunofluorescence combination was based on confocal images of at least 3 independent embryoids for each time point.

**Knockdown of SOX17 in TCam-2**

TCam-2 cell line was kindly provided by Professor Sohei Kitazawa and Janet Shipley and was maintained in advanced RPMI 1640 (GIBCO, 12633) supplemented with 10% fetal bovine serum, 100U/ml Penicillin-0.1 mg/ml Streptomycin and 2mM L-Glutamine.

To suppress the expression of SOX17 in TCam-2, we used inducible expression vector containing miRNA against SOX17 into TCam-2. microRNA (miR) were designed by BLOCK-iT RNAi Designer (http://rnaidesigner.lifetechnologies.com/rnaiexpress/). miR sequence for SOX17 miR-1 were Fw: 5′- TTCAAATTCCGTGCGGTCCACGTTTTGGCCACTGACTGACGTGGACCGCGGAATTTGAA-3′; for SOX17 miR-2 were Fw: 5′- TGCAGATACTGTTCAAATTCCGTTTTGGCCACTGACTGACGGAATTTGCAGTATCTGCA-3′. As a control, non-targeting miR was previously designed (Hackett et al., 2013). These miRs were cloned into a vector downstream of a tetracycline response element (TRE). The vectors were co-transfected with vector containing the reverse tetracycline transactivator (rtTA) with Venus, a gene encoding a variant of yellow fluorescent protein. Transfected TCam-2 were cultured 3 days in the presence of doxycycline (Sigma). After culture, Venus positive cells were collected by S3 Cell Sorter (Bio-Rad) and resuspended in lysis buffer for RNA extraction.

**Western Blot Analysis**

Whole-cell extracts were prepared by sorted cells from day 4 embryoids (WT; TNAP/NANOS3-mCherry double positive population, BLIMP1 KO; TNAP positive population, SOX17 KO; whole population) in lysis buffer composed of 50mM Tris-HCl (pH7.5), 0.15M NaCl, 0.1% SDS, 1% Triton X-100, 1% Sodium deoxycholate and cOmplete mini EDTA free (Roche Applied Science, Penzberg, Germany). After electrophoresis, proteins were transferred to nitrocellulose membranes. Membranes were incubated in Western Blocking Reagent (Roche Applied Science) and treated with antibodies. Primary antibodies against BLIMP1 (rat IgG; eBioscience), SOX17 (goat IgG; R&D systems), and TUBULIN (mouse IgG; Sigma) were used. Horseradish peroxidase-conjugated secondary antibodies against rat, goat or rat IgG were added (Dako, Life technologies). After antibody treatment, blots were developed using ECL Western Blotting Detection System (GE Healthcare).

## Author Contributions

The study was conceived and designed by N.I., L.W., J.H.H., and M.A.S. The NANOS3-mCherry reporter hESC lines and the BLIMP1 and SOX17 knockout hESCs were generated by L.W. and S.V. hESC growth conditions were developed by L.W. and J.H.H. The PGCLC induction experiments were performed by N.I. and L.W. W.W.C.T. collected human embryos and performed immunofluorescence, RNA-seq, and bioinformatics analysis, together with S.D. and Y.M. Experiments on TCam-2, including the knockdowns, exogenous SOX17 expression experiments, and western blot analysis, were performed by T.K. The study was supervised by M.A.S. and J.H.H. The manuscript was written by N.I., W.W.C.T., J.H.H., and M.A.S. with input from most authors.

## Accession Numbers

The NCBI GEO accession number for the RNA-seq data reported in this paper is GSE60138.

## Supplemental Information

Table S1. Transcript Expression Levels in Replicates of Indicated Samples by RNA-Seq, Related to Figure 2

Table S2. Transcript Expression Levels in Replicates of WIS2 hESC Cultured in 4i and Conventional Conditions, Related to Figure 7

Table S3. Primers for RT-qPCR, Related to Figures 1, 3, 5, 6, 7, and S6

Document S1. Article plus Supplemental Information
