# Supplementary material for: SOX17 Is a Critical Specifier of Human Primordial Germ Cell Fate
Source: Cell. 2015 Jan 15;160(1-2):253–68. doi: 10.1016/j.cell.2014.12.013 (PMC4310934; doi:10.1016/j.cell.2014.12.013)
Supplement: Table S3. Primers for RT-qPCR, Related to Figures 1, 3, 5, 6, 7, and S6 [file mmc3.pdf]

Table S3. Primers for RT-qPCR, Related to Figures 1, 3, 5, 6, 7, and S6

Target		5’ → 3’
β-ACTIN	Forward	ATAGCAACGTACATGGCTGG
	Reverse	CACCTTCTACAATGAGCTGC
GAPDH	Forward	CGCTTCGCTCTCTGCTCCTCCTGT
	Reverse	GGTGACCAGGCGCCCAATACGA
mCherry	Forward	GATAACATGGCCATCATCAAGGA
	Reverse	CGTGGCCGTTCACGGAG
TNAP	Forward	AAGCAGGTCTTGGGGTGCACCA
	Reverse	TTGGTCTCGCCAGTACTTGGGGT
NANOS3	Forward	CCCGAAACTCGGCAGGCAAGA
	Reverse	AAGGCTCAGACTTCCCGGCAC
KIT	Forward	TGCATTCAAGCACAATGGCACGG
	Reverse	GTGTGGGGATGGATTTGCTCTTTGT
BLIMP1	Forward	CGGGGAGAATGTGGACTGGGTAGAG
	Reverse	CTGGAGTTACACTTGGGGGCAGC
TFAP2C	Forward	CGCTCATGTGACTCTCCTGACATCC
	Reverse	TGGGCCGCCAATAGCATGTTCT
OCT4	Forward	GCTGGAGCAAAACCCGGAGG
	Reverse	TCGGCCTGTGTATATCCCAGGGTG
NANOG	Forward	TGCTGAGATGCCTCACACGGA
	Reverse	TGACCGGGACCTTGTCTTCCTT
STELLA	Forward	ACGCCGATGGACCCATCACAGTTT
	Reverse	TCTCGGAGGAGATTTGAGAGGCCC
PRDM14	Forward	CTACCGAGCCCGAGTGGCCTAC
	Reverse	TAGAGCCATCCCGGGACCGCA
SOX2	Forward	ACACCAA TCCCA TCCACACT
	Reverse	CCTCCCCAGGTTTTCTCTGT
SOX17	Forward	GAGCCAAGGGCGAGTCCCGTA
	Reverse	CCTTCCACGACTTGCCCAGCAT
HOXA1	Forward	CGGGAACCTGGGGGTGTCCTAC
	Reverse	TTCTTGGTGGGTCTGCTTCCTGA
MYC	Forward	GGACCCGCTTCTCTGAAAGG
	Reverse	TAACGTTGAGGGGCATCGTC
T (BRACHYURY)	Forward	ACCCAGTTCATAGCGGTGAC
	Reverse	CCATTGGGAGTACCCAGGTT
HOXB1	Forward	GCGAGCTTTGCACCGGCCTA
	Reverse	ACCTTCGCTGTCTTGGGTGGGT
GATA6	Forward	CCCACAACACAACCTACAGC
	Reverse	GCGAGACTGACGCCTATGTA
DND1	Forward	TGCTGGGACAGGGACCTATG
	Reverse	ACGGCCATGGAAGATCACTG
KLF4	Forward	TACCAAGAGCTCATGCCACC
	Reverse	CGCGTAATCACAAGTGTGGG

UTF1	Forward	GACCAGCTGCTGACCTTGAAC
	Reverse	CCAGGGACACTGTCTGGTCG
HOXC4	Forward	ATCTCTCCCCACCCCTATCG
	Reverse	TGATCACGGGGCATTTCACA
MIXL1	Forward	TACCCCGTCTCTTCAACCCT
	Reverse	GCATGCAGAGTCATTGGAGC
PDGFRA	Forward	CTCCCTGGCTGTTCTGATCG
	Reverse	TGCCAACCCTGTTCCAAAGT
EOMES	Forward	CTGGCTTCCGTGCCCACGTC
	Reverse	CATGCGCCTGCCCTGTTTCG
CDX2	Forward	TCACCATCCGGAGGAAAGCC
	Reverse	CTCTCCTTTGCTCTGCGGTT
KDR	Forward	ACATTCAGCTCAAGGCTCCC
	Reverse	TGAACCTCCCGCATTCAGTC
GATA4	Forward	TCCCTCTTCCCTCCTCAAAT
	Reverse	TCAGCGTGTAAAGGCATCTG
FOXA1	Forward	AAGGCATACGAACAGGCACTG
	Reverse	TACACACCTTGGTAGTACGCC
HNF1B	Forward	CAATCCACTCTCAGGAGGCG
	Reverse	ATCGTGGGAGAGGCATTGTG
HNF4A	Forward	GGCAATGTGTCAGGGAGGAA
	Reverse	CAGGGATTTCAGGGGCACTT
